# Musculoskeletal disorders: does cuproptosis hold the key?

**DOI:** 10.3389/fcell.2026.1790043

**Published:** 2026-05-20

**Authors:** Chenglong Wang, Dengshang Liu, Yu Ye, Liqiang Cui, Shuangquan Gong, Hongjun Liu, Shiming Xie

**Affiliations:** Spinal Surgery Department, Mianyang Orthopaedic Hospital, Mianyang, Sichuan, China

**Keywords:** cuproptosis, cuproptosis-related genes, intervertebral disc degeneration, musculoskeletal disorders, osteoarthritis, osteoporosis, rheumatoid arthritis

## Abstract

Cuproptosis, a recently identified form of programmed cell death, is triggered by intracellular copper accumulation and executed through mitochondrial metabolic interference and proteostasis disruption. While emerging evidence has elucidated its pathophysiological roles in oncology, neurodegenerative disorders, and metabolic diseases, its mechanistic involvement and therapeutic potential in musculoskeletal disorders-including osteoarthritis, rheumatoid arthritis, osteoporosis, intervertebral disc degeneration, ankylosing spondylitis, bone tumors, skeletal muscle atrophy, and osteonecrosis of the femoral head (ONFH)-remain largely unexplored. This comprehensive review synthesizes current knowledge on the molecular mechanisms underlying cuproptosis in musculoskeletal pathophysiology, its potential as a disease-modifying factor, and the translational challenges that must be addressed before its clinical application. Furthermore, we highlight critical knowledge gaps and propose novel research directions that may help advance this emerging field.

## Introduction

Cell death primarily encompasses programmed cell death (such as apoptosis, pyroptosis, ferroptosis) and accidental cell death (necrosis), each exhibiting distinct morphological, mechanistic, and functional characteristics. These diverse modes collectively contribute to development, immune responses, and disease pathogenesis (Bertheloot et al., 2021; Tong et al., 2022). Recently, researchers have identified a novel cell death pathway triggered by aberrant intracellular copper ion accumulation, termed cuproptosis (Tsvetkov et al., 2022). Cuproptosis has been implicated as a potential pathogenic mechanism underlying several neurodegenerative disorders, including Alzheimer’s disease (AD) and Parkinson’s disease (PD). In these conditions, dysregulated copper ions may drive neuronal death by promoting β-amyloid (Aβ) aggregation in AD or exacerbating α-synuclein toxicity in PD. Mechanistically, copper ions likely induce neurodegenerative damage by altering the folding dynamics of these pathological proteins, accelerating their oligomerization, and amplifying oxidative stress-mediated cellular injury (Li X. et al., 2023; Huang et al., 2024). Elevated intracellular copper concentrations are a hallmark of malignant cells, correlating closely with their heightened metabolic demands for rapid proliferation. This essential transition metal orchestrates pivotal oncogenic processes, including angiogenesis, metastatic dissemination, and uncontrolled mitotic expansion (Xie et al., 2023). While copper overload activates pro-survival redox signaling pathways that foster neoplastic progression, its inherent cytotoxicity can paradoxically trigger oxidative catastrophe and cuproptosis-mediated tumor suppression (Wang M. et al., 2023). Despite growing recognition of cuproptosis in neurodegenerative and oncological pathologies, its implications in musculoskeletal disorders-a system encompassing bones, joints, skeletal muscles, and associated connective tissues-remain conspicuously understudied. While initial research has focused on bone and joint pathologies, emerging evidence also points to a role for cuproptosis in skeletal muscle and bone vascular diseases (Li X. et al., 2023; Huang et al., 2024; Xie et al., 2023). This review systematically synthesizes current evidence, identifies critical knowledge gaps, and discusses the potential therapeutic paradigms that could arise from targeting copper-dependent cell death mechanisms, while acknowledging that clinical translation remains at an early stage. To move beyond disease-specific descriptions, this review also synthesizes shared mechanistic themes-including copper homeostasis dysregulation, mitochondrial metabolic convergence, and redox imbalance-and distinguishes disease-specific divergences in pathological context.

### Cuproptosis

**FIGURE 1:**
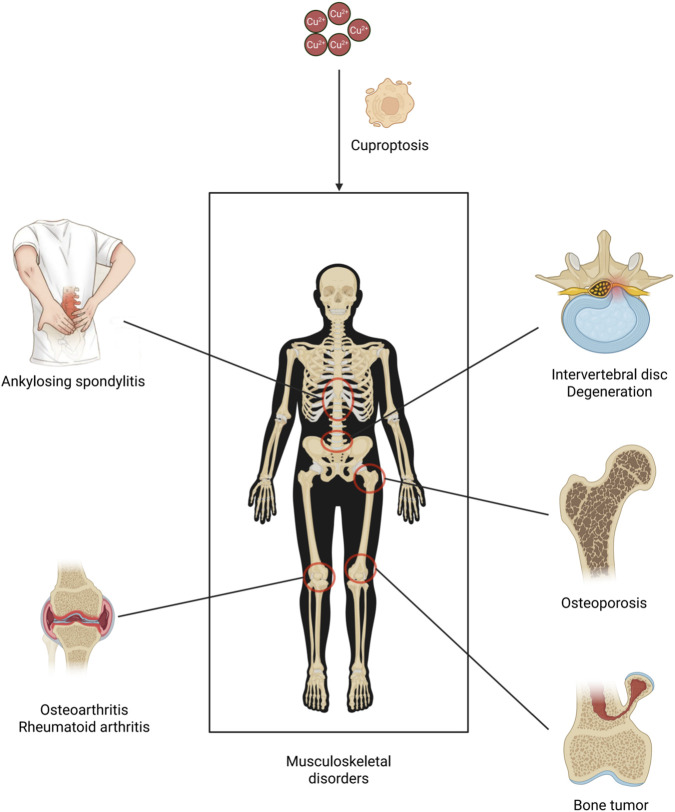
Copper overload induces cuproptosis, a regulated cell death mechanism triggered by intracellular copper accumulation, which contributes to the pathogenesis of multiple musculoskeletal disorders through dysregulation of copper homeostasis. These conditions include osteoarthritis, rheumatoid arthritis, osteoporosis, intervertebral disc degeneration, ankylosing spondylitis, and bone tumors (Created by the Biorender).

Cuproptosis is a newly identified form of programmed cell death mediated by copper ions, characterized primarily by aberrant mitochondrial copper accumulation and its interaction with key metabolic enzymes. In a landmark 2022 Science study, Tsvetkov et al. systematically elucidated the molecular mechanism of cuproptosis, demonstrating that copper ions selectively bind to lipoylated proteins in the tricarboxylic acid (TCA) cycle (such as DLAT and DLST). This interaction induces protein oligomerization, impairs mitochondrial function, and ultimately triggers cell death (Tsvetkov et al., 2022). Cuproptosis is triggered by intracellular copper accumulation, a process primarily mediated by the copper transporter protein 1 (CTR1/SLC31A1). Following cellular uptake, copper ions are reduced to Cu^+^ by ferredoxin 1 (FDX1), whose activity further governs the stability of lipoic acid (LA)-modified TCA cycle enzymes (Tsvetkov et al., 2022). Notably, studies demonstrate that both copper ionophores (e.g., elesclomol) and depletion of intracellular glutathione (GSH) can potentiate copper accumulation, thereby exacerbating cuproptosis (Xie et al., 2023). The core mechanism of cuproptosis involves direct binding of copper ions to lipoylated mitochondrial proteins (e.g., DLAT, DLST, and the pyruvate dehydrogenase complex), triggering their aberrant aggregation and functional impairment (Tsvetkov et al., 2022). Furthermore, copper overload disrupts the stability of iron-sulfur (Fe-S) cluster proteins, exacerbating damage to the mitochondrial electron transport chain (ETC.) and energy metabolism (Ling et al., 2025). These cumulative effects induce proteotoxic stress, manifesting as characteristic morphological hallmarks of cell death, including mitochondrial cristae disruption, loss of plasma membrane integrity, and chromatin condensation (Tang et al., 2022). The discovery of cuproptosis has unveiled novel therapeutic perspectives for diverse diseases. Notably, in oncology, cuproptosis may emerge as a promising anti-tumor strategy, as certain malignant cells exhibit heightened susceptibility to copper-induced cell death (Xie et al., 2023). Beyond oncology, dysregulated copper metabolism has been implicated in cardiovascular and neurodegenerative disorders, suggesting that modulating cuproptosis could yield broad clinical applications (Wang D. et al., 2023). Nevertheless, the precise regulatory networks governing cuproptosis and its pathophysiological roles remain to be fully elucidated, necessitating further investigation. Of particular interest is its potential implication in musculoskeletal disorders, although research in this area remains in its nascent stages (Figure 1).

## Therapeutic strategies based on cuproptosis in musculoskeletal diseases

### Osteoarthritis and rheumatoid arthritis

Osteoarthritis (OA) and rheumatoid arthritis (RA) represent two clinically and etiologically distinct joint disorders. OA is primarily characterized by progressive cartilage degeneration, osteophyte formation, and chronic pain, predominantly affecting weight-bearing joints (such as knees and hips) in elderly populations (Glyn-Jones et al., 2015). In contrast, RA constitutes a systemic autoimmune disorder featuring symmetric synovitis, joint destruction, and systemic inflammation, typically accompanied by morning stiffness and positive rheumatoid factor (Smolen et al., 2016). Epidemiologically, OA demonstrates an age-dependent prevalence, affecting approximately 7% of the global population, with incidence rates escalating markedly with advancing age. In contrast, RA exhibits a lower overall prevalence (0.5%–1% worldwide), yet displays a distinct female predominance. Both conditions impose substantial functional impairment, significantly compromising patients’ quality of life while creating considerable socioeconomic burdens (Glyn-Jones et al., 2015; Smolen et al., 2016). Current therapeutic strategies encompass both pharmacological and non-pharmacological interventions. OA management primarily relies on analgesics (NSAIDs), intra-articular injections (corticosteroids or hyaluronic acid), and joint replacement surgery (Glyn-Jones et al., 2015). Conversely, RA treatment focuses on immunomodulators (DMARDs) and biologic agents (TNF-α inhibitors) to suppress inflammatory progression (Glyn-Jones et al., 2015). However, these approaches present significant limitations: OA therapies provide only symptomatic relief without halting cartilage degeneration, while RA medications may induce infections, drug resistance, or impose substantial financial burdens, with suboptimal responses in certain patient subsets. Furthermore, the underlying pathogenesis of both diseases remains incompletely understood, particularly the lack of targeted therapies for OA.

This therapeutic impasse underscores the urgent need for mechanistic investigations into disease pathogenesis. The recent discovery of novel cell death modalities, particularly cuproptosis, has provided transformative insights for arthritis research. Emerging evidence suggests that dysregulated copper metabolism may critically intersect with oxidative stress, inflammatory signaling activation, and articular tissue damage, potentially serving as a key driver of chondrocyte apoptosis in OA, and a fundamental mechanism underlying synovial hyperplasia in RA (Yu et al., 2024; Han et al., 2024). Elucidating these pathways may help overcome some current therapeutic limitations and might inform the development of novel strategies, such as tissue-specific copper chelators with tissue-specific delivery, precision modulators of copper transport systems, and small molecules targeting copper-dependent signaling networks. Such mechanistic breakthroughs may ultimately improve clinical outcomes while alleviating the global burden of these debilitating conditions.

Copper dysregulation has been implicated in the pathogenesis of both OA and RA, though its mechanistic roles differ. In OA, serum and synovial fluid copper levels are elevated compared to healthy controls (Yang et al., 2023; Yazar et al., 2005), with higher concentrations correlating with increased arthritis risk (Guan et al., 2023). Similarly, RA patients exhibit marked increases in synovial fluid and plasma copper (Yazar et al., 2005), alongside distinctive features such as blue albumin accumulation and an altered copper/ceruloplasmin ratio (Scudder et al., 1978). While the exact contributions of copper to disease progression remain incompletely understood, therapeutic strategies targeting copper homeostasis show promise in both conditions. In OA, copper-based interventions leverage its redox-active properties: synthetic copper complexes induce senescent chondrocyte apoptosis via reactive oxygen species (ROS) -mediated mechanisms, promoting cartilage regeneration (Zhou et al., 2021), while copper-integrated nanoparticles enhance chondrocyte function in tissue engineering (Ghanbari et al., 2021). A thermosensitive hydrogel (HPP@Cu gel) further demonstrates multifunctional efficacy by scavenging reactive species, polarizing macrophages toward an anti-inflammatory M2 phenotype, and mitigating cartilage degradation (Zhu et al., 2022). Conversely, RA research has focused on modulating copper-dependent pathways-such as hirudin’s inhibition of ceruloplasmin degradation, which reduces synovial inflammation (Sokolov et al., 2015)-and optimizing chelation therapy (d-penicillamine) (Pasqualicchio et al., 1996). Preclinical studies underscore copper’s anti-inflammatory effects in both OA and RA models, highlighting its dual role as a pathogenic driver and therapeutic target (Pasqualicchio et al., 1996; Omoto et al., 2005).

Emerging evidence suggests that cuproptosis-related genes (CRGs) may contribute to the pathogenesis of OA and RA, with bioinformatic analyses revealing distinct expression patterns that potentially link them to synovial inflammation, cartilage degradation, and metabolic dysregulation. In OA, CRGs exhibit tissue-specific dysregulation: synovial tissues show elevated FDX1, LIPT1, PDHA1, PDHB, DLAT, and CDKN2A alongside reduced DBT, GLS, and DLST, while cartilage demonstrates upregulation of MTF1 and CDKN2A, the latter driving chondrocyte senescence (Wang et al., 2023c; Kim et al., 2014; Nong et al., 2023; Zhou et al., 2004; Meyer et al., 2021). Notably, GLS correlates with immune activation, implicating CRGs in synovitis progression (Chang et al., 2023). In RA, CRG expression varies by tissue: synovial fibroblasts upregulate DLST, DLD, and ATP7A but downregulate PDHB, PDHA1, LIAS, DLAT, and CDKN2A, whereas blood samples exhibit elevated FDX1, LIAS, LIPT1, DLD, and CDKN2A with reduced MTF1-discrepancies potentially attributable to sample-type differences (Wang A. et al., 2023). Functionally, CRGs modulate disease mechanisms through metabolic and inflammatory pathways. In OA, MTF1 links synovitis to cartilage degradation (Kim et al., 2014), while in RA, DLAT promotes chondrocyte death and cartilage damage (Hu et al., 2023a). CRGs further influence RA pathogenesis via glycolysis (PDHA1, PDHB, GLS1, MTF1 in fibroblast-like synoviocytes), oxidative stress (LIAS), mitochondrial function (DLAT), and immune dysregulation (CDKN2A-mediated cytokine release and T/B-cell disruption) (Zhao et al., 2022). These findings suggest that CRGs may contribute to joint pathology and represent potential therapeutic targets that warrant further investigation (Table 1).

**TABLE 1:** Cuproptosis-related genes (CRGs) in osteoarthritis (OA) and rheumatoid arthritis (RA).

Gene	Main functions/Mechanisms	Expression pattern	Key findings/References
FDX1	Regulates copper reduction and TCA cycle enzyme lipoylation; mediates oxidative stress-induced cuproptosis	Upregulated in OA synovium	Correlates with synovial inflammation and cartilage degradation (Wang et al., 2023c; Chang et al., 2023)
LIPT1	Involved in lipoic acid metabolism and mitochondrial energy production	Upregulated in OA synovium	Linked to immune activation and metabolic dysregulation in OA (Wang et al., 2023c; Nong et al., 2023)
PDHA1	Modulates glycolysis and inflammatory responses	Upregulated in OA synovium; Downregulated in RA synovium	Promotes chondrocyte senescence in OA (Wang et al., 2023c) Suppressed in RA synovial fibroblasts (Wang et al., 2023d)
PDHB	Maintains mitochondrial homeostasis and inhibits ERK signaling	Upregulated in OA synovium; Downregulated in RA synovium	Inhibits osteoclast differentiation in OA; dysregulated in RA pathogenesis (Wang et al., 2023c; Wang et al., 2023d)
DLAT	Mediates pyruvate-to-acetyl-CoA conversion; triggers TCA cycle enzyme aggregation	Upregulated in OA synovium; Downregulated in RA synovium	Contributes to cartilage degradation in OA and RA (Wang et al., 2023c; Hu et al., 2023a)
CDKN2A	Promotes cellular senescence and cytokine release	Upregulated in OA cartilage and RA blood	Drives chondrocyte senescence in OA; correlates with immune dysregulation in RA (Wang et al., 2023c; Wang et al., 2023d)
GLS	Regulates glutamine metabolism and antioxidant activity	Downregulated in OA synovium	Depletion suppresses cuproptosis, impairing osteoblast function (Zhao et al., 2022; Biltz et al., 1983)
MTF1	Maintains metal ion homeostasis and redox balance	Upregulated in OA cartilage	Links synovitis to cartilage degradation in OA (Kim et al., 2014)
ATP7A	Mediates copper efflux; regulates intracellular copper levels	Upregulated in RA synovium	Associated with synovial inflammation and oxidative stress in RA (Wang et al., 2023d)

Current CRG signatures in OA and RA exhibit notable inconsistencies. DLAT is reported as upregulated in OA synovium (Wang et al., 2023c) but downregulated in RA synovial fibroblasts (Wang A. et al., 2023); CDKN2A expression patterns vary by tissue type across studies (Wang et al., 2023c; Wang A. et al., 2023). These discrepancies likely stem from differences in sample source and disease context.

Crucially, only MTF1 has received robust wet-lab validation for its role in OA chondrocytes (Kim et al., 2014). The remaining genes—despite plausible links to mitochondrial metabolism—lack protein-level or functional evidence confirming their direct involvement in cuproptosis. For example, changes in GLS or LIAS mRNA could reflect general metabolic shifts rather than a specific cuproptotic process. Thus, these signatures should be regarded as hypothesis-generating tools, not validated biomarkers.

**FIGURE 2:**
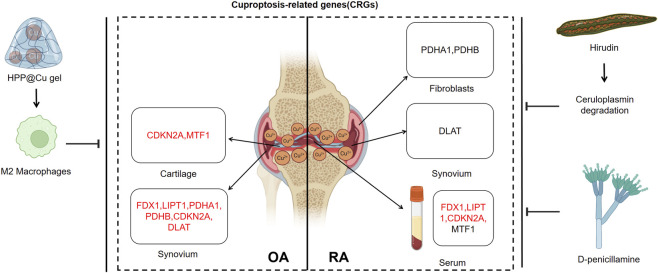
Expression profiles of cuproptosis-related genes exhibit distinct patterns across osteoarthritis (OA) and rheumatoid arthritis (RA) tissues, with red and black indicating high and low expression levels, respectively. Therapeutic strategies targeting cuproptosis-including copper-incorporated hydrogels, hirudin, and D-penicillamine-demonstrate promising potential for OA and RA treatment (Created by the Biorender).

While the involvement of CRGs in RA and OA pathogenesis remains incompletely characterized, emerging evidence suggests their potential therapeutic relevance. In RA, CRGs appear to contribute to disease mechanisms, as indirectly supported by the historical use of copper chelation therapy - though its clinical application requires optimization. Similarly in OA, CRGs demonstrate importance in disease pathways, yet their precise roles demand further clinical investigation. Elucidation of copper-dependent pathways in both conditions may uncover novel molecular mechanisms underlying disease progression, potentially yielding biomarkers or therapeutic strategies for synovitis management (Figure 2).

### Osteoporosis

Bone homeostasis is regulated by the equilibrium between osteoclast-driven bone resorption and osteoblast-mediated bone formation (Compston et al., 2019). An imbalance in this process-whether due to excessive resorption or insufficient formation-leads to reduced bone mass and microarchitectural deterioration, ultimately contributing to osteoporosis (OP) (Compston et al., 2019; Boyle et al., 2003). Osteoporotic fractures pose a significant health risk, particularly in elderly populations, and carry considerable socioeconomic consequences (Taipaleenmäki et al., 2022). Current OP therapies primarily target either the suppression of osteoclast activity or the stimulation of bone formation (Cui et al., 2021). While osteoclast inhibitors-including estrogen, bisphosphonates, denosumab, and calcitonin-have long been used clinically, their application is limited by adverse effects (Cui et al., 2021; Brown, 2017). Similarly, bone-forming agents such as parathyroid hormone and strontium ranelate present challenges, with side effects ranging from nausea to leg cramps upon prolonged use (Oryan and Sahvieh, 2021). Thus, identifying novel therapeutic agents and uncovering their mechanisms of action remain critical for advancing OP treatment.

The human body contains approximately 50–120 mg of copper, with skeletal muscles and bones harboring about 60% of this content (Olivares and Uauy, 1996; Turnlund et al., 1998). A study of 728 postmenopausal women (aged 45–80 years) revealed significantly lower serum copper levels in OP individuals than in healthy controls, with a positive correlation between copper concentrations and bone mineral density (BMD) (Okyay et al., 2013). Consistent with these findings, other studies identified an inverse relationship between serum copper levels and OP risk (Arikan et al., 2011; Gür et al., 2002). Additionally, higher dietary and total copper intake was associated with increased BMD and reduced OP incidence in adults (Fan et al., 2022).

**FIGURE 3:**
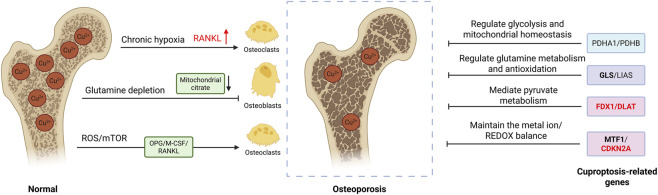
Illustrates: (i) the differential expression of copper ions in normal versus osteoporotic (OP) tissues; (ii) expression patterns of cuproptosis-related genes in OP, with red indicating high expression levels and black denoting lower expression; and (iii) mechanisms by which distinct patterns of cuproptosis-related genes mediate copper-dependent cell death in OP pathogenesis (Created by the Biorender).

As a vital micronutrient, copper plays a crucial role in cellular energy metabolism by serving as a key component of oxidase enzymes involved in electron transfer during oxygen reduction (Rondanelli et al., 2021) (Figure 3). Notably, lysyl oxidase-a copper-dependent monoamine oxidase-catalyzes the cross-linking of collagen and elastin through its action on lysine and hydroxylysine residues, which is essential for connective tissue formation (Institute of Medicine US Panel on Micronutrients, 2001; Dahl et al., 2005; Rucker et al., 1998). Copper also functions as an indispensable cofactor for numerous metalloenzymes participating in collagen biosynthesis (Pepa and Brandi, 2016). Insufficient copper levels may disrupt bone homeostasis and contribute to osteoporotic conditions (Qu et al., 2018). *In vitro* experiments demonstrate copper’s capacity to modulate bone metabolism by enhancing mesenchymal stem cell differentiation toward osteogenic lineages (Ding et al., 2014). Additionally, copper-containing antioxidant enzymes protect bone tissue by scavenging free radicals and stimulating osteoblast function (Zofková et al., 2013). These effects exhibit dose-dependency, with low concentrations supporting osteoblast proliferation while excessive amounts prove cytotoxic (Milkovic et al., 2014). Notably, research by Li et al. revealed that copper deficiency may impair superoxide dismutase activity, potentially leading to enhanced osteoclast function and accelerated bone resorption (Rondanelli et al., 2021).

Pathogenic studies indicate that ROS play a critical role in OP development (Manolagas, 2010). Although bone and marrow exist in a hypoxic state, hypoxia exerts a protective effect against ROS-mediated bone damage (Yellowley and Genetos, 2019). Osteoblasts and osteoclasts-oxygen-sensitive regulators of bone homeostasis-exhibit remodeling dynamics modulated by hypoxia (Arnett et al., 2003; Stegen et al., 2018). However, chronic hypoxia upregulates RANKL, promoting osteoclastogenesis (Zhu et al., 2019). OP pathogenesis involves marrow microenvironmental shifts and disrupted cellular homeostasis, processes potentially tied to cuproptosis-related metabolism (Zhao et al., 2022). Glutamine, essential for osteoblast energy metabolism (Biltz et al., 1983), supports mitochondrial citrate production in precursors (Karner et al., 2015) and is required for matrix mineralization (Brown et al., 2011). Its depletion may suppress cuproptosis, influencing osteoblast function. Hypoxia, while potentially inhibiting cuproptosis (Tsvetkov et al., 2022), disrupts copper dynamics by impairing antioxidant defenses and exacerbating ROS-mediated copper cytotoxicity (Pan et al., 2020). ROS-driven oxidative stress indirectly regulates osteoclast activity via osteoprotegerin, M-CSF, and RANKL signaling, while mTOR-dependent glycolysis and glutamine metabolism in effector T cells may further suppress cuproptosis (Zhao et al., 2022).

Li et al. described in detail several cuproptosis key genes related to the pathogenesis of OP (Table 2). PDHA1 modulates glycolysis and inflammatory responses, while PDHB suppresses ERK signaling and maintains mitochondrial homeostasis, potentially influencing osteoclast differentiation. GLS primarily regulates glutamine metabolism and antioxidant activity. LIAS plays a crucial role in mitochondrial energy metabolism, particularly in oxidative stress and inflammation. DLAT may contribute to OP by mediating pyruvate-to-acetyl-CoA conversion in the pyruvate dehydrogenase complex and mitochondrial metabolism. FDX1 could impact OP through metabolic pathways, immune cell regulation, immune-related gene expression, and inflammatory processes. CDKN2A is implicated in cellular senescence, whereas DLD influences OP via antioxidant effects, cellular energy production, and bone integration. MTF1 may directly or indirectly affect OP by maintaining metal ion homeostasis, redox balance, and gene expression regulation, while also protecting against metal toxicity. LIPT1 might participate in OP through its role in the tricarboxylic acid cycle and mitochondrial energy metabolism (Li D. et al., 2023).

**TABLE 2:** Cuproptosis-related genes (CRGs) in osteoporosis (OP).

Gene	Main functions/Mechanisms	Expression pattern	Key findings/References
PDHA1	Modulates glycolysis and inflammatory responses	-	Affects osteoclast differentiation via metabolic reprogramming (Li et al., 2023b)
PDHB	Suppresses ERK signaling; maintains mitochondrial stability	-	Inhibits osteoclastogenesis by stabilizing mitochondrial function (Li et al., 2023b)
GLS	Regulates glutamine metabolism; supports matrix mineralization	Downregulated	Glutamine depletion impairs osteoblast energy metabolism and cuproptosis (Zhao et al., 2022; Li et al., 2023b)
LIAS	Participates in oxidative stress and mitochondrial energy metabolism	-	Associated with ROS-mediated bone resorption (Li et al., 2023b)
DLAT	Mediates pyruvate dehydrogenase activity; disrupts mitochondrial metabolism	Upregulated	Promotes mitochondrial dysfunction and bone loss (Li et al., 2023b)
FDX1	Regulates immune cell activity and metabolic pathways	Upregulated	Linked to immune-metabolic crosstalk in bone remodeling (Li et al., 2023b)
CDKN2A	Induces cellular senescence	Upregulated	Accelerates osteoblast aging and bone microarchitectural deterioration (Li et al., 2023b)
DLD	Antioxidant effects; supports cellular energy production	-	Protects osteoblasts from oxidative damage (Li et al., 2023b)
MTF1	Maintains copper homeostasis and redox balance	Downregulated	Copper deficiency exacerbates osteoclast activation (Li et al., 2023b)
LIPT1	Involved in TCA cycle and mitochondrial function	-	Dysregulation impairs osteoblast differentiation (Li et al., 2023b)

### Intervertebral disc degeneration

Intervertebral Disc Degeneration (IDD) represents a prevalent degenerative spinal disorder characterized by the depletion of nucleus pulposus (NP) cells, degradation of extracellular matrix (ECM), and structural disruption of the annulus fibrosus (AF) (Wang C. et al., 2022). These pathological changes ultimately compromise the disc’s biomechanical functionality, leading to clinical manifestations including chronic low back pain and nerve root compression. Epidemiological studies indicate that approximately 540 million individuals worldwide suffer from low back pain, with IDD contributing to nearly 40% of these cases, thereby imposing substantial socioeconomic burden (GBD, 2021 Low Back Pain Collaborators, 2023; Hartvigsen et al., 2018). Although the precise pathogenic mechanisms underlying IDD remain incompletely understood, accumulating evidence underscores the pivotal role of programmed cell death pathways-including apoptosis, necroptosis, pyroptosis, and ferroptosis-in driving disc degeneration progression (Wang C. et al., 2022).

Cuproptosis contributes to enzyme and protein synthesis and modulates cellular signaling pathways. Its homeostasis is tightly regulated by copper proteases, chaperone proteins, and membrane transporters. Dysregulated copper metabolism can disrupt intracellular osmotic balance, induce metabolic dysfunction, impair cell signaling, and promote DNA damage (Wang Y. et al., 2022). In 2022, Tsvetkov et al. demonstrated that cuproptosis directly targets lipoylated proteins in the TCA cycle, triggering their aggregation and downregulating iron-sulfur cluster proteins, ultimately inducing proteotoxic stress and cuproptosis (Tsvetkov et al., 2022; Chen et al., 2022). Elevated serum copper levels have been observed in individuals with IDD, correlating positively with Pfirrmann’s classification (Staszkiewicz et al., 2023). Lysyl oxidase (LOX), a copper-dependent amine oxidase, catalyzes the cross-linking of collagen and elastin to stabilize the ECM through the insolubilization of ECM proteins. LOX expression in NP cells demonstrates a negative correlation with both Pfirrmann grade and patient age. Notably, both protein and mRNA expression levels of LOX in NP tissue exhibit a progressive decline with advancing intervertebral disc (IVD) degeneration (Chen et al., 2025). Study identified eight cuproptosis-related regulators. Validation experiments revealed that the expression levels of CRGs (FDX1, LIAS, LIPT1, GCSH, DLST, DLAT, and PDHB) were significantly downregulated in NP samples from IDD patients compared with controls. Conversely, ATP7A, ATP7B, and MTF1 exhibited upregulated expression patterns. These findings suggest that these CRGs may serve as potential biomarkers for IDD (Zhang et al., 2023). Notably, MTF1 is highly expressed in degenerative discs, particularly in NP cells, suggesting its potential role in IDD pathogenesis (Hu Y. et al., 2023) (Table 3).

**TABLE 3:** Cuproptosis-related genes (CRGs) in intervertebral disc degeneration (IDD).

Gene	Main functions/Mechanisms	Expression pattern	Key findings/References
FDX1	Mediates copper reduction and TCA cycle enzyme aggregation	Downregulated	Oxidative stress upregulates FDX1, inducing cuproptosis in NP cells (Zhang et al., 2023; Chen et al., 2024)
LIAS	Participates in mitochondrial oxidative phosphorylation	Downregulated	Loss correlates with reduced antioxidant capacity in IDD (Zhang et al., 2023)
LIPT1	Facilitates lipoic acid-dependent enzyme activity	Downregulated	Impaired mitochondrial metabolism accelerates disc degeneration (Zhang et al., 2023)
DLST	Catalyzes α-ketoglutarate conversion in TCA cycle	Downregulated	Reduced expression disrupts energy metabolism in NP cells (Zhang et al., 2023)
DLAT	Mediates pyruvate dehydrogenase activity; triggers cuproptosis	Downregulated	Aggregation of DLAT exacerbates mitochondrial dysfunction (Zhang et al., 2023)
ATP7A	Regulates copper efflux	Upregulated	Compensatory upregulation under chronic copper overload (Zhang et al., 2023)
MTF1	Maintains metal ion homeostasis	Upregulated	High expression correlates with advanced disc degeneration (Hu et al., 2023b)
PDHB	Supports mitochondrial energy production	Downregulated	Loss contributes to metabolic insufficiency in NP cells (Zhang et al., 2023)

Emerging evidence reveals crosstalk between ferroptosis and cuproptosis, particularly in mitochondrial metabolism, GSH regulation, and oxidative stress (Liu and Chen, 2024). The TCA cycle influences GSH synthesis and lipid peroxide accumulation, contributing to both ferroptosis and cuproptosis. However, the exact mechanism of DLAT in this process remains unclear (Tsvetkov et al., 2022). Bioinformatic analysis implicates miR-15a-5p as a potential regulator of cuproptosis-related genes in the TCA cycle (Li Y. et al., 2023). Pharmacologically, compounds such as tretinoin and penicillamine have been shown to modulate cuproptosis *in vitro* (Wang et al., 2023e); whether repurposing these copper-regulating drugs can mitigate IDD progression requires dedicated *in vivo* investigation.

Elevated ferredoxin-1 (FDX1) levels were observed in degenerated intervertebral discs from rats and humans. In NP cells, sublethal oxidative stress upregulated FDX1, triggering lipoylation and aggregation of TCA cycle proteins and inducing cell death upon physiological Cu^2+^ exposure-effects mitigated by FDX1 knockdown. Oxidative stress elevated CTR1 and ATP7A expression, and CTR1 inhibition reduced TCA protein aggregation and cell death. Mechanistically, oxidative stress increased specificity protein 1 (SP1), which promoted CTR1 transcription. SP1 suppression not only attenuated cell death but also preserved disc hydration and reduced tissue degeneration. These results demonstrate that oxidative stress drives FDX1 upregulation and copper influx via SP1-dependent CTR1 activation, disrupting the TCA cycle and inducing cuproptosis (Chen et al., 2024).

A study developed multifunctional PG@Cu-FP metal-polyphenol nanoparticles that effectively target mitochondria to scavenge excess reactive oxygen species. These nanoparticles suppressed NOD-like receptor family pyrin domain containing 3 (NLRP3) inflammasome activation by inhibiting Gasdermin D oligomerization, consequently downregulating NOD-like receptor expression. *In vivo* studies demonstrated PG@Cu-FP’s efficacy in maintaining intervertebral disc height, hydration, and tissue integrity (Zhou et al., 2024).

Bioactive hydrogels offer a promising approach to modulate cellular behavior and maintain tissue homeostasis. Another research engineered a dynamic hydrogel (HA-NCSN/Cu) via reductive chelation between thiourea-grafted hyaluronic acid (HA-NCSN) and Cu^2+^. HA-NCSN/Cu efficiently scavenges ROS, mitigating inflammatory stress in NP cells. RNA-seq reveals significant regulation of the glutathione pathway, while HA-NCSN/Cu activates the TGF-β/Smad pathway, enhancing Aggrecan and Collagen II production. By synergistically suppressing inflammation and promoting extracellular matrix regeneration, the hydrogel showed promise in restoring structure and function in a pre-clinical model of degenerated intervertebral discs, as demonstrated *in vivo* (Bu et al., 2025).

IDD exemplifies the gap between bioinformatic prediction and mechanistic reality. Zhang et al. identified multiple CRGs as downregulated in NP tissue (Zhang et al., 2023), seemingly at odds with a cuproptosis-driven death model. However, Chen et al. subsequently resolved this paradox through wet-lab experiments, demonstrating that oxidative stress upregulates SP1, which activates CTR1 transcription, increases copper influx, and drives FDX1-dependent DLAT aggregation (Chen et al., 2024). Genetic silencing of FDX1 or CTR1 abolished this effect.

This case highlights a critical limitation of transcript-level bioinformatics: mRNA expression does not necessarily correlate with the post-translational events—namely protein lipoylation and aggregation—that execute cuproptosis. Therefore, future biomarker efforts in IDD must incorporate protein-level assays.

**FIGURE 4:**
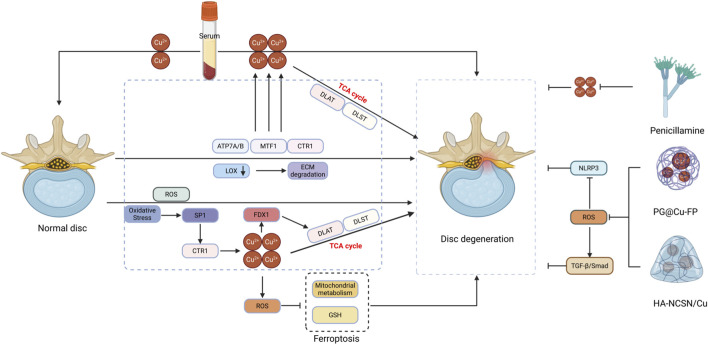
Illustrates: (i) the differential copper levels between normal and degenerated intervertebral discs, (ii) distinct patterns of copper-induced cell death and their underlying molecular mechanisms, and (iii) therapeutic strategies for disc degeneration targeting cuproptosis, including copper-incorporated hydrogels, copper-based nanoparticles, and penicillamine (Created by the Biorender).

In summary, cuproptosis represents a distinct cell death pathway that may contribute to IDD pathogenesis (Figure 4). Current evidence is limited to serological associations, with no direct histopathological correlation between copper levels and IDD. The molecular mechanisms remain poorly understood, warranting further investigation. Future studies should clarify cuproptosis’s role in IDD and explore therapeutic strategies, including copper chelators and nanoparticle-based delivery systems, potentially in combination with other cell death regulators.

### Ankylosing spondylitis

Ankylosing spondylitis (AS) is a chronic inflammatory disorder predominantly affecting the axial skeleton, with a marked male predominance (male-to-female ratio-3:1). The disease typically manifests in early adulthood, with peak onset occurring between 20 and 30 years of age (Mau et al., 2021). Diagnostic delays, averaging 6–7 years, frequently result in severe spinal fusion, joint damage, and significant disability (Walsh and Magrey, 2021). Diagnosis relies on clinical evaluation, HLA-B27 testing (positive in ∼90% of cases), and imaging (e.g., MRI for early sacroiliitis). Treatment includes NSAIDs, disease-modifying agents (sulfasalazine), and biologics (anti-TNFα, IL-17 inhibitors), which significantly improve symptoms but remain costly. Severe cases may require joint replacement (Walsh and Magrey, 2021). Despite therapeutic advances, challenges include high costs of biologics, incomplete disease control, and irreversible structural damage in late-stage AS.

**FIGURE 5:**
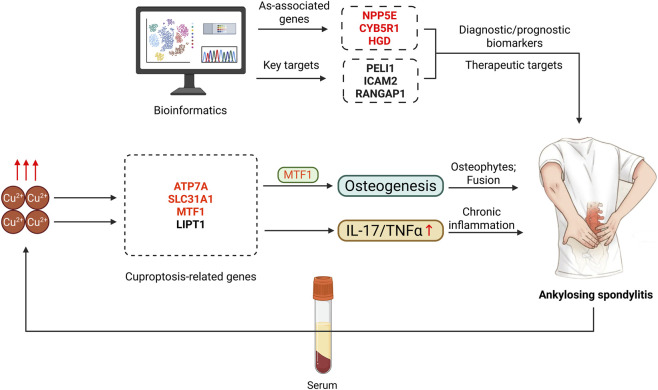
Demonstrates that serum copper levels are elevated in ankylosing spondylitis (AS) patients. The expression patterns of cuproptosis-related genes in AS are displayed, with red and black indicating high and low expression levels, respectively (consistent throughout subsequent panels). Bioinformatics-based predictions identify key cuproptosis-associated genes and potential therapeutic targets in AS. Furthermore, the schematic illustrates putative mechanisms through which cuproptosis-related genes may contribute to AS pathogenesis (Created by the Biorender).

Recent studies implicate cuproptosis, a copper-dependent cell death mechanism, in AS pathogenesis (Figure 5). Elevated copper levels and dysregulated CRGs (such as ATP7A, MTF1) are observed in AS patients, potentially influencing osteogenesis and immune dysregulation (Zhang P. et al., 2024). Targeting cuproptosis pathways represents a speculative but potentially rewarding area for discovering novel biomarkers or therapies; however, current evidence is purely computational, and any clinical potential remains entirely unproven. Bioinformatic analyses identified three genes (INPP5E, CYB5R1, and HGD) associated with AS (Fan J. et al., 2024). Furthermore, an independent bioinformatic study predicted eight cuproptosis-related prognostic regulators (LIPT1, DLD, PDHA1, PDHB, SLC31A1, ATP7A, MTF1, and CDKN2A) for AS risk stratification. Peripheral blood analysis revealed significantly elevated expression levels of key CRG (ATP7A, MTF1, and SLC31A1) in AS cases compared to controls, whereas LIPT1 exhibited an inverse expression pattern. Notably, bioinformatic analyses indicate a correlation between high MTF1 expression and osteogenic potential, although the functional significance of this association in AS pathogenesis is not yet known. This analysis pinpointed critical CRGs-PELI1, ICAM2, and RANGAP1-as pivotal regulators in AS progression (Wei et al., 2025). Current research efforts, primarily focused on bioinformatic analyses and serological investigations (Table 4), remain limited in scope.

**TABLE 4:** Cuproptosis-related genes (CRGs) in ankylosing spondylitis (AS).

Gene	Main functions/Mechanisms	Expression pattern	Key findings/References
ATP7A	Mediates copper transport and homeostasis	Upregulated	Correlates with elevated serum copper levels and osteogenic potential (Zhang et al., 2024a)
MTF1	Regulates metal-responsive gene expression	Upregulated	Enhances osteogenesis and immune dysregulation in AS (Zhang et al., 2024a)
SLC31A1	Copper transporter (CTR1); facilitates copper uptake	Upregulated	Promotes intracellular copper accumulation, driving inflammation (Zhang et al., 2024a)
LIPT1	Involved in mitochondrial lipoylation	Downregulated	Reduced expression linked to metabolic dysfunction in AS (Zhang et al., 2024a)
CDKN2A	Induces cellular senescence and cytokine release	Upregulated	Associated with chronic inflammation and tissue fibrosis (Zhang et al., 2024a)
PELI1	Modulates NF-κB signaling and immune responses	Upregulated	Promotes inflammatory cytokine production (Wei et al., 2025)
ICAM2	Mediates leukocyte adhesion and immune cell infiltration	Upregulated	Contributes to synovial inflammation and osteogenesis (Wei et al., 2025)
RANGAP1	Regulates nucleocytoplasmic transport and immune activation	Upregulated	Linked to HLA-B27-associated pathogenesis (Wei et al., 2025)

Evidence for cuproptosis in AS rests entirely on computational analyses of peripheral blood, without any tissue-level or functional validation. Moreover, independent studies have identified completely non-overlapping gene sets: an eight-gene panel from Zhang et al. (Zhang P. et al., 2024) versus PELI1, ICAM2, RANGAP1 from Wei et al. (2025). This fundamental lack of consensus indicates that results are highly sensitive to model parameters and that a true cuproptosis signature in AS has not been defined. All current findings should be considered preliminary explorations requiring direct investigation of sacroiliac and spinal tissues. More comprehensive studies are urgently needed to elucidate the underlying mechanisms of cuproptosis in AS and to explore novel therapeutic strategies.

### Bone tumour

Bone tumors comprise heterogeneous neoplasms arising from osseous tissue or metastatic deposits, clinically manifesting as localized pain, pathological fractures, swelling, and impaired mobility (Global Burden of Disease, 2019 Cancer Collaboration et al., 2022). Malignant subtypes (such as osteosarcoma (OS), chondrosarcoma) may exhibit systemic symptoms such as weight loss and fatigue (Beird et al., 2022). Although primary bone tumors represent only 0.2% of malignancies-with OS predominating in pediatric populations and chondrosarcoma in older adults-metastatic skeletal involvement is prevalent in advanced breast and prostate cancers (Choi and Ro, 2021). Despite their rarity, these tumors impose disproportionate clinical burdens: aggressive growth patterns, high disability rates, and metastatic potential severely compromise patient quality of life (Global Burden of Disease, 2019 Cancer Collaboration et al., 2022). Pediatric cases face developmental disruptions, while metastatic disease strains healthcare systems. Current management combines surgical resection with adjuvant therapies (neoadjuvant chemotherapy for OS; denosumab for giant cell tumors) (Yu and Yao, 2024). Persistent challenges include surgical morbidity, chemoresistance, radiotherapy toxicity, and limited efficacy of targeted agents. Prognosis remains poor for advanced disease, as evidenced by OS’s metastatic 5-year survival plummeting from 60% to 70% to <20% (Beird et al., 2022).

**FIGURE 6:**
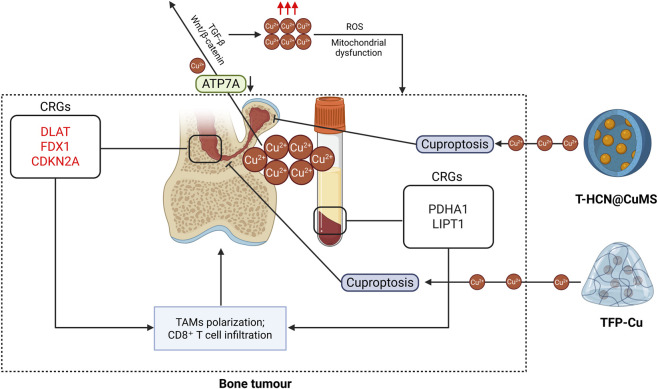
Presents (i) elevated copper levels in both tissue and serum samples from bone tumor patients; (ii) the potential mechanisms through which differentially expressed cuproptosis-related genes (highlighted in red for upregulated and black for downregulated expressions) contribute to bone tumor progression; and (iii) the utilization of copper-containing hydrogels and copper-based nanomaterials to deliver copper ions that induce tumor cell cuproptosis (Created by the Biorender).

Emerging insights into cuproptosis reveal its dual role in tumor proliferation and therapeutic resistance, suggesting novel intervention strategies (Figure 6). However, the mechanistic interplay between cuproptosis and the bone tumor microenvironment requires clarification. Multidisciplinary integration of multi-omics, advanced organoid models, and translational research is critical to overcome current therapeutic limitations. Dysregulated copper homeostasis has been implicated in OS pathogenesis. Elevated serum copper levels have been observed in OS patients, whereas those who underwent amputation and remained tumor-free exhibited copper concentrations comparable to healthy controls (Breiter et al., 1978). Therapeutically, copper alloys demonstrate favorable properties for post-resection recovery, including blood compatibility, antimicrobial activity, and osteogenic potential (Wang et al., 2021; Lin J. et al., 2020). In chemical dynamic therapy (CDT), Cu^2+^ acts as a catalyst, depleting GSH via redox reactions to amplify CDT efficacy (Chu et al., 2023). At concentrations of 25–100 μM, Cu^2+^ compounds selectively impair OS cell viability, with pronounced effects on malignant osteoblasts relative to normal cells (León et al., 2014). Certain copper complexes further exhibit reduced cytotoxicity toward healthy osteoblasts while maintaining potent anti-OS activity (Cadavid-Vargas et al., 2019). Although excessive copper accumulation may exacerbate toxicity in OS cells-which inherently harbor high copper levels-these findings underscore the therapeutic potential of copper-based strategies. However, the translational impact of modulating copper levels during disease progression remains unexplored, warranting further investigation.

Bioinformatic studies have explored whether CRGs might serve as candidate biomarkers for diagnosis, immune microenvironment characterization, and prognosis in OS. However, the proposed signatures vary widely and have not been validated in independent clinical cohorts (Table 5). Studies indicate that certain genes-particularly DLAT, FDX1, and CDKN2A-are significantly upregulated in OS tissues, whereas ATP7A expression is reduced. The expression patterns of PDHA1 and LIPT1 vary across sample types, such as blood and osteoblasts, necessitating further investigation. Evidence also links CRGs to OS progression via immune cell modulation and cancer-related pathways (Yang M. et al., 2022; Wang X. et al., 2022; Jiang et al., 2023). For instance, researchers identified PDHA1 and CDKN2A as early diagnostic markers with high predictive efficacy (Jiang et al., 2023), while Li et al. reported a positive correlation between CDKN2A protein levels and Ki67, suggesting its potential as a therapeutic target (Li et al., 2022).

**TABLE 5:** Cuproptosis-related genes (CRGs) in bone tumors (osteosarcoma, OS).

Gene	Main functions/Mechanisms	Expression pattern	Key findings/References
DLAT	Triggers TCA cycle enzyme aggregation and cuproptosis	Upregulated	Aggregation promotes mitochondrial proteotoxicity (Yang et al., 2022a; Feng et al., 2023)
FDX1	Mediates copper reduction and oxidative stress	Upregulated	High expression correlates with poor prognosis and metastasis (Feng et al., 2023; Hu et al., 2023c)]
CDKN2A	Promotes tumor cell proliferation and immune evasion	Upregulated	Positively correlates with Ki67 index; potential therapeutic target (Jiang et al., 2023; Li et al., 2022)
PDHA1	Modulates glycolysis and TGF-β/Wnt signaling	-	Identified as an early diagnostic marker (Jiang et al., 2023)
LIPT1	Supports mitochondrial lipoylation and energy metabolism	-	Downregulation linked to immune suppression (Yang et al., 2022b)
ATP7A	Regulates copper efflux; confers drug resistance	Downregulated	Loss increases intracellular copper, sensitizing cells to cuproptosis (Li et al., 2018)
LIAS	Participates in antioxidant defense and iron-sulfur cluster synthesis	Downregulated	Low expression correlates with immune cell exclusion (Yang et al., 2022b)
MTF1	Maintains copper homeostasis and redox balance	-	Dual role in tumor suppression and progression (Yang et al., 2022a; Hu et al., 2023c)

*In vitro* evidence suggests that FDX1 overexpression enhances OS cell migration. Therefore, strategies aimed at inhibiting FDX1 merit further study as a potential therapeutic approach, although its *in vivo* efficacy and safety are completely unknown (Feng et al., 2023). Conversely, LIAS and PDK1 exhibit negative correlations with immune cell infiltration (Yang W. et al., 2022). Additionally, CRG upregulation activates pro-tumorigenic pathways, including TGF-β, Wnt/β-catenin, and p53 signaling, which may be therapeutically targeted to impede OS progression (Han et al., 2022; Matsuoka et al., 2020; Hu et al., 2023c). Prognostic analyses further associate LIAS, PDK1, and cuproptosis-related lncRNAs with OS outcomes (Hu et al., 2023c; Ni et al., 2023; Liu et al., 2022; Huang et al., 2023), while elevated FDX1 levels correlate with poorer prognosis, underscoring its utility as a prognostic indicator (Hu et al., 2023c). A large number of studies have constructed different risk models, which can effectively predict the CRGs or cuproptosis-related long non-coding RNAs (lncRNAs) related to OS prognosis, and some lncRNAs have also been verified by experiments (Feng et al., 2023; Jiang et al., 2022; Jia et al., 2024; Gao Z. et al., 2025; Li et al., 2024). Collectively, CRGs or cuproptosis-related lncRNAs provide critical insights into OS diagnosis, immune regulation, and clinical outcomes, aiding therapeutic decision-making. However, these results lack OS tumor prognostic specificity, specifically, each study identified/predicted different biomarkers. And the mechanistic role of cuproptosis in immune evasion remains unclear, warranting deeper exploration of these genes in OS pathogenesis and treatment.

Recent study developed a two-dimensional nanomaterial, T-HCN@CuMS, based on heterogeneous carbon nitride (HCN) and copper-loaded molybdenum disulfide (CuMS). By integrating multiple therapeutic mechanisms-including photocatalytic ROS generation, Fenton-like reactions, and cuproptosis induction-the nanosystem demonstrated significantly enhanced antitumor and antimetastatic efficacy under near-infrared (NIR) irradiation. Its therapeutic potential was further validated in a high-metastatic orthotopic osteosarcoma model, offering a promising multimodal strategy for cancer therapy using rationally designed 2D nanomaterials (Xia et al., 2023). Furthermore, Xie et al. designed an injectable TFP-Cu gel, composed of Cu^2+^-coordinated tremella fuciformis polysaccharide, which smartly releases Cu^2+^ in response to the tumor microenvironment, selectively inhibiting osteosarcoma proliferation (via cell cycle arrest, apoptosis, and cuproptosis) while promoting M1 macrophage polarization, immune modulation, and osteogenic activity. In murine models, TFP-Cu significantly suppressed tumor growth and enhanced CD8^+^ T-cell responses, offering a multifunctional biomaterial strategy for osteosarcoma therapy (Xie et al., 2024). Another study developed an alginate-based hydrogel co-encapsulating Cu-Fe3O4 nanozymes and artemisinin, which achieves sustained release of nanoparticles and drugs to synergistically amplify oxidative stress via ROS accumulation and carbon radicals, while concurrently triggering ferroptosis (through GPX4 inhibition) and cuproptosis (via DLAT pathway activation). The system demonstrated potent tumor-killing efficacy and excellent biocompatibility *in vitro* and *in vivo* (Zhang Y. et al., 2024).

The OS literature contains the largest number of cuproptosis-related predictive models, yet no two models share the same core gene set. One study emphasizes FDX1, LIAS, PDK1 (Hu et al., 2023c), while others focus on distinct lncRNAs (Liu et al., 2022; Huang et al., 2023) or DLAT, FDX1, CDKN2A (Jiang et al., 2023). This extreme heterogeneity undermines reproducibility.

A deeper issue is specificity: CDKN2A is a canonical senescence regulator, and PDHA1 is a glycolysis enzyme. Their association with OS transcriptomes may reflect general tumor metabolism rather than cuproptosis *per se*. By contrast, the most compelling therapeutic evidence comes not from these gene signatures, but from independent pharmacological studies showing that copper-based nanomaterials induce *bona fide* DLAT aggregation and mitochondrial damage in OS models (Xia et al., 2023; Xie et al., 2024). Reconciling disparate bioinformatic models against such direct mechanistic evidence is a priority for the field.

### Skeletal muscle atrophy

Skeletal muscle atrophy, encompassing conditions such as sarcopenia, disuse atrophy, and cachexia, is characterized by progressive loss of muscle mass and function (Nunes et al., 2022). Copper is essential for skeletal muscle physiology, serving as a cofactor for the antioxidant enzyme SOD1 and for cytochrome c oxidase in mitochondrial respiration (Kim et al., 2008). Genetic evidence further underscores this importance: mutations in the copper transporter ATP7A cause Menkes disease, which prominently features severe muscle hypotonia (Kaler, 2011).

The core machinery of cuproptosis-FDX1-mediated protein lipoylation and subsequent aggregation of TCA cycle enzymes-is fundamentally a mitochondrial proteotoxic stress pathway (Li et al., 2025). Given that mitochondrial dysfunction is a hallmark of muscle atrophy, it is plausible to hypothesize that age- or disease-related dysregulation of copper homeostasis (e.g., altered CTR1 or ATP7A expression) could sensitize myofibers to cuproptosis, contributing to muscle degeneration (Li et al., 2025).

Despite this theoretical framework, direct evidence linking cuproptosis to muscle atrophy is virtually absent. No study has yet profiled CRGs at the protein level in atrophic human muscle. While altered serum copper levels have been sporadically reported in sarcopenic individuals, the findings are inconsistent in direction and confounded by systemic inflammation (Yan et al., 2025). Notably, satellite cells-the muscle stem cells responsible for regeneration-are highly dependent on mitochondrial metabolism for activation and differentiation (Bhattacharya and Scimè, 2020; Voshtani et al., 2025). It is therefore conceivable that aberrant copper accumulation within the satellite cell niche could impair regenerative capacity, although this remains entirely speculative.

In summary, whether cuproptosis contributes to skeletal muscle atrophy is an open question. Future studies should prioritize measuring copper content and key cuproptosis markers in atrophic versus healthy human muscle, and testing whether modulation of copper transporters influences muscle wasting in preclinical models. Given the urgent need for novel therapeutic targets in sarcopenia and cachexia, this line of inquiry merits dedicated investigation despite its currently preliminary status.

### Osteonecrosis of the femoral head

Osteonecrosis of the femoral head (ONFH) is characterized by ischemia-induced death of osteocytes and marrow elements, ultimately leading to femoral head collapse (Petek et al., 2019). The ischemic microenvironment of ONFH raises an intriguing but entirely unexplored question regarding cuproptosis (Gao S.H. et al., 2025). Since hypoxia was shown to inhibit cuproptosis by dampening mitochondrial respiration (Yoshioka and Lee, 2013), it remains unknown whether the low-oxygen milieu of the necrotic femoral head suppresses cuproptosis in surviving bone cells, or whether ischemia–reperfusion injury instead triggers copper-mediated toxicity upon reoxygenation.

To date, no studies have profiled copper levels or cuproptosis-related gene expression in ONFH tissues. Given the established role of oxidative stress and mitochondrial dysfunction in ONFH pathogenesis, and the dependence of cuproptosis on these same pathways, investigating copper dyshomeostasis in this context represents a logical but wholly unexplored research direction.

### Shared and disease-specific mechanisms of cuproptosis in musculoskeletal disorders

While the preceding sections have examined cuproptosis in individual musculoskeletal diseases, a synthesis of shared mechanistic themes and disease-specific divergences is essential for conceptual coherence. Across the disorders discussed, three unifying principles emerge: dysregulation of copper homeostasis, convergence on mitochondrial metabolism, and interplay with oxidative stress and hypoxia.

### Shared mechanisms

Copper homeostasis dysregulation. In virtually all musculoskeletal disorders reviewed, copper homeostasis is disrupted-though the directionality differs. Serum and local tissue copper levels are elevated in OA, RA, IDD, AS, and OS, suggesting a common theme of copper overload in inflammatory and neoplastic conditions (Yazar et al., 2005; Staszkiewicz et al., 2023; Zhang P. et al., 2024; Breiter et al., 1978). Conversely, osteoporosis is characterized by systemic copper deficiency, which impairs LOX-dependent collagen crosslinking and antioxidant defenses (Okyay et al., 2013). This bidirectional dysregulation underscores the narrow therapeutic window of copper: both excess and deficiency can compromise tissue integrity, albeit through distinct downstream mechanisms.

Mitochondrial metabolism as a common target. The cuproptosis execution machinery-FDX1-mediated protein lipoylation and aggregation of TCA cycle enzymes (DLAT, DLST)-represents a convergent node across diseases. In IDD, direct experimental evidence demonstrates that oxidative stress-driven FDX1 upregulation and SP1/CTR1-mediated copper influx trigger DLAT aggregation and NP cell death (Chen et al., 2024). In OS, copper-based nanomaterials induce *bona fide* cuproptosis via the same FDX1-DLAT axis, confirming that this core pathway is functional in musculoskeletal cells (Xia et al., 2023; Zhang Y. et al., 2024). Bioinformatic analyses further implicate TCA cycle-related CRGs (PDHA1, PDHB, GLS, LIAS) in OA, RA, and OP, although the specific contribution of protein lipoylation versus general metabolic reprogramming requires experimental clarification.

Oxidative stress and hypoxia interplay. Copper is both an antioxidant cofactor (via SOD1) and a potent pro-oxidant in excess (via Fenton-like reactions) (Han et al., 2024; Rondanelli et al., 2021). This dual nature places copper at the center of redox imbalance in musculoskeletal diseases. In OA and IDD, elevated copper amplifies ROS production, contributing to chondrocyte and NP cell death (Yu et al., 2024; Han et al., 2024; Chen et al., 2024). Hypoxia adds another layer of complexity: while acute hypoxia inhibits cuproptosis by dampening mitochondrial respiration (Tsvetkov et al., 2022), chronic hypoxia-as in the bone marrow microenvironment or ONFH-disrupts copper dynamics, potentially sensitizing cells to cuproptosis upon reoxygenation (Pan et al., 2020; Gao SH. et al., 2025; Yoshioka and Lee, 2013). The ROS-copper-hypoxia axis thus represents a unifying, albeit context-dependent, pathogenic mechanism.

### Disease-specific divergences

Despite these shared themes, the pathological consequences of copper dyshomeostasis vary by tissue and disease context (Table 6).

**TABLE 6:** Shared and disease-specific features of cuproptosis across musculoskeletal disorders.

Disease	Copper status	Key CRG/Mechanism	Primary target cell	Dominant context
OA	Synovial/serum Cu↑	MTF1, FDX1, CDKN2A	Chondrocyte	Degeneration + Mechanical stress
RA	Synovial/plasma Cu↑	DLST, DLD, ATP7A	Synoviocyte, Immune cell	Autoimmune inflammation
OP	Serum Cu↓	PDHA1, GLS, FDX1	Osteoblast/Osteoclast	Metabolic deficiency
IDD	Serum Cu↑	FDX1, SP1/CTR1 axis, DLAT	Nucleus pulposus cell	Oxidative stress + Mechanical load
AS	Serum Cu↑	ATP7A, MTF1, SLC31A1	Osteoblast, Immune cell	Inflammation + Pathological ossification
OS	Tissue/serum Cu↑	DLAT, FDX1, CDKN2A	Tumor cell	Malignant proliferation
Muscle Atrophy	Unknown	SLC25A12, PABPC4	Myofiber, Satellite cell	Metabolic/Disuse degeneration
ONFH	Unknown	Unknown	Osteocyte	Ischemia-hypoxia

Degenerative vs. inflammatory contexts. In OA and IDD, copper-mediated damage primarily targets structural cells (chondrocytes, NP cells) within mechanically stressed, relatively avascular tissues. In RA and AS, autoimmune inflammation adds a layer of immune cell-mediated copper toxicity. The same CRG (e.g., MTF1) may thus link to cartilage catabolism in OA but to immune dysregulation in RA.

Metabolic vs. proliferative demands. In OP, copper deficiency compromises osteoblast function and collagen quality, representing a hypometabolic, deficiency-driven pathology (Okyay et al., 2013; Fan et al., 2022; Rondanelli et al., 2021; Rucker et al., 1998; Qu et al., 2018). In OS, copper addiction fuels malignant proliferation, and cuproptosis induction becomes a therapeutic opportunity (Xie et al., 2023; Breiter et al., 1978; Xia et al., 2023; Xie et al., 2024; Zhang Y. et al., 2024). These opposing contexts-deficiency versus addiction-illustrate why the same pathway must be therapeutically modulated in opposite directions.

Tissue-specific drug delivery challenges. Avascular cartilage in OA and IDD limits the penetration of copper-based nanotherapeutics, whereas the highly vascularized tumor microenvironment in OS facilitates nanoparticle accumulation (Xia et al., 2023; Shi et al., 2023; Wang et al., 2024). These physical differences necessitate tissue-specific delivery strategies.

Taken together, the current evidence supports a model wherein copper dyshomeostasis-whether overload or deficiency-converges on mitochondrial metabolism to influence cell fate in musculoskeletal tissues. The pathological outcome (matrix degradation, immune activation, bone loss, or tumor progression) is dictated by tissue context, disease-specific stressors, and the direction of copper imbalance. Future mechanistic studies should move beyond correlating CRG expression with disease to directly measuring copper distribution, protein lipoylation status, and FDX1 enzymatic activity across tissue types and disease stages.

### Prospect and challenge

The discovery of cuproptosis, a novel copper-dependent form of regulated cell death driven by mitochondrial proteotoxic stress (Tsvetkov et al., 2022), has opened new conceptual avenues for understanding musculoskeletal disorders and has sparked interest in potential therapeutic applications. As highlighted in the preceding synthesis, the convergence of these disorders on copper homeostasis and mitochondrial metabolism suggests that certain therapeutic strategies-such as FDX1 modulation or tissue-specific copper chelation-may have cross-disease applicability. However, translating these insights into treatments for OA, OP, OS, or muscle atrophy remains a distant goal. While its therapeutic potential is compelling, significant challenges remain in elucidating its mechanistic specificity, achieving copper homeostasis, and translating findings into clinical applications.

### Mechanistic complexity and technical hurdles

Cuproptosis intersects with other cell death pathways, particularly ferroptosis and apoptosis, complicating its isolation as a distinct process. Unlike ferroptosis, which hinges on iron-mediated lipid peroxidation, cuproptosis is characterized by copper-induced aggregation of lipoylated TCA cycle enzymes (e.g., DLAT) and FDX1-dependent mitochondrial dysfunction (Liu and Chen, 2024). However, overlapping features-such as ROS accumulation and mitochondrial collapse-demand advanced tools for differentiation. For instance, spatial metabolomics (DESI-MSI) and copper-specific biosensors (FRET-based probes) could delineate copper’s spatiotemporal dynamics in tissues (Fan X. et al., 2024; Melnychuk et al., 2020). Moreover, the dual role of copper in musculoskeletal physiology-supporting collagen crosslinking via LOX while triggering cytotoxicity at excess levels-necessitates precise modulation to avoid disrupting tissue repair (Lin W. et al., 2020; Rodriguez-Pascual and Rosell-Garcia, 2018).

### Disease-specific heterogeneity and therapeutic dilemmas

The context-dependence of cuproptosis poses another challenge. In OA, synovial fluid copper overload promotes chondrocyte death, yet systemic copper chelation risks impairing LOX-dependent cartilage integrity (Han et al., 2024; Kaler, 2011). Conversely, osteosarcoma cells, despite their reliance on CTR1-mediated copper uptake, are vulnerable to cuproptosis inducers like disulfiram-copper complexes (Mandell et al., 2019). However, ATP7B-mediated copper efflux in tumor cells may foster resistance (Li et al., 2018). Similarly, in muscle atrophy, cuproptosis inhibition could preserve mitochondrial function but might inadvertently suppress satellite cell activation (Tsitkanou et al., 2016). These nuances underscore the need for tissue-targeted strategies, such as pH-responsive nanoparticles or exosome-delivered miRNAs to regulate copper transporters (e.g., ATP7A/B) (Yan et al., 2020; Gu et al., 2025; Sun et al., 2024).

### Clinical translation: balancing efficacy and safety

Current barriers to clinical adoption include drug toxicity and inadequate delivery systems. For example, while gold-copper nanoparticles enhance osteosarcoma targeting, their poor penetration into avascular cartilage limits OA applications (Shi et al., 2023; Wang et al., 2024). Emerging solutions include biomimetic mineralized nanoparticles for bone-specific copper release and engineered exosomes to modulate copper metabolism genes (Bozorgi et al., 2022; Sun et al., 2025). Future trials must also validate biomarkers-such as serum copper-to-ceruloplasmin ratios or mitochondrial ROS signatures-to stratify patients likely to benefit from cuproptosis modulation.

## Conclusion

Translating cuproptosis insights into musculoskeletal therapies will demand a multidisciplinary approach, integrating mechanistic insights (e.g., FDX1 inhibition), tissue-specific delivery technologies, and rigorous clinical validation. Addressing these challenges may eventually unlock targeted strategies, especially for cancers like osteosarcoma, but achieving systemic safety and disease-specific efficacy remains a formidable task. Over the next decade, rigorous collaborative efforts spanning molecular biology, nanotechnology, and clinical trial design will be essential to determine whether the concept of cuproptosis can be successfully translated from bench to bedside.

## References

[B1] Arikan D. C. Coskun A. Ozer A. Kilinc M. Atalay F. Arikan T. (2011). Plasma selenium, zinc, copper and lipid levels in postmenopausal Turkish women and their relation with osteoporosis. Biol. Trace Elem. Res. 144 (1-3), 407–417. 10.1007/s12011-011-9109-7 21656042

[B2] Arnett T. R. Gibbons D. C. Utting J. C. Orriss I. R. Hoebertz A. Rosendaal M. (2003). Hypoxia is a major stimulator of osteoclast formation and bone resorption. J. Cell. Physiol. 196 (1), 2–8. 10.1002/jcp.10321 12767036

[B3] Beird H. C. Bielack S. S. Flanagan A. M. Gill J. Heymann D. Janeway K. A. (2022). Osteosarcoma. Nat. Rev. Dis. Prim. 8 (1), 82. 10.1038/s41572-022-00416-z 36585406

[B4] Bertheloot D. Latz E. Franklin B. S. (2021). Necroptosis, pyroptosis and apoptosis: an intricate game of cell death. Cell. Mol. Immunol. 18 (5), 1106–1121. 10.1038/s41423-020-00630-3 33785842 PMC8008022

[B5] Bhattacharya D. Scimè A. (2020). Mitochondrial function in muscle stem cell fates. Front. Cell. Dev. Biol. 8, 480. 10.3389/fcell.2020.00480 32612995 PMC7308489

[B6] Biltz R. M. Letteri J. M. Pellegrino E. D. Palekar A. Pinkus L. M. (1983). Glutamine metabolism in bone. Min. Electrolyte Metab. 9 (3), 125–131. 6135980

[B7] Boyle W. J. Simonet W. S. Lacey D. L. (2003). Osteoclast differentiation and activation. Nature 423 (6937), 337–342. 10.1038/nature01658 12748652

[B8] Bozorgi A. Mozafari M. Khazaei M. Soleimani M. Jamalpoor Z. (2022). Fabrication, characterization, and optimization of a novel copper-incorporated chitosan/gelatin-based scaffold for bone tissue engineering applications. Bioimpacts 12 (3), 233–246. 10.34172/bi.2021.23451 35677664 PMC9124876

[B9] Breiter D. N. Diasio R. B. Neifeld J. P. Roush M. L. Rosenberg S. A. (1978). Serum copper and zinc measurement in patients with osteogenic sarcoma. Cancer 42 (2), 598–602. 10.1002/1097-0142(197808)42:2<598::aid-cncr2820420228>3.0.co 307981

[B10] Brown C. (2017). Osteoporosis: staying strong. Nature 550 (7674), S15–S17. 10.1038/550S15a 28976955

[B11] Brown P. M. Hutchison J. D. Crockett J. C. (2011). Absence of glutamine supplementation prevents differentiation of murine calvarial osteoblasts to a mineralizing phenotype. Calcif. Tissue Int. 89 (6), 472–482. 10.1007/s00223-011-9537-6 21972050

[B12] Bu P. Peng R. Zhang J. He Z. Gou S. Liu X. (2025). A one-stone-two-birds strategy for intervertebral disc repair: constructing a reductive chelation hydrogel to mitigate oxidative stress and promote disc matrix reconstruction. Adv. Mater. 37 (6), e2411290. 10.1002/adma.202411290 39713901

[B13] Cadavid-Vargas J. F. Arnal P. M. Mojica Sepúlveda R. D. Rizzo A. Soria D. B. Di Virgilio A. L. (2019). Copper complex with sulfamethazine and 2,2'-bipyridine supported on mesoporous silica microspheres improves its antitumor action toward human osteosarcoma cells: Cyto- and genotoxic effects. Biometals 32 (1), 21–32. 10.1007/s10534-018-0154-y 30334122

[B14] Chang B. Hu Z. Chen L. Jin Z. Yang Y. (2023). Development and validation of cuproptosis-related genes in synovitis during osteoarthritis progress. Front. Immunol. 14, 1090596. 10.3389/fimmu.2023.1090596 36817415 PMC9932029

[B15] Chen L. Min J. Wang F. (2022). Copper homeostasis and cuproptosis in health and disease. Signal Transduct. Target Ther. 7 (1), 378. 10.1038/s41392-022-01229-y 36414625 PMC9681860

[B16] Chen X. Li K. Xiao Y. Wu W. Lin H. Qing X. (2024). SP1/CTR1-mediated oxidative stress-induced cuproptosis in intervertebral disc degeneration. Biofactors 50 (5), 1009–1023. 10.1002/biof.2052 38599595

[B17] Chen C. Feng W. Sun Z. Lv L. Lin C. Lin D. (2025). Relationships between intervertebral disc degeneration and lysyl oxidase expression in human nucleus pulposus. Biomed. Rep. 22 (5), 84. 10.3892/br.2025.1962 40151800 PMC11948296

[B18] Choi J. H. Ro J. Y. (2021). The 2020 WHO classification of tumors of bone: an updated review. Adv. Anat. Pathol. 28 (3), 119–138. 10.1097/PAP.0000000000000293 33480599

[B19] Chu X. Zhang L. Li Y. He Y. Zhang Y. Du C. (2023). NIR responsive doxorubicin-loaded hollow copper ferrite @ polydopamine for synergistic Chemodynamic/photothermal/chemo-therapy. Small 19 (7), e2205414. 10.1002/smll.202205414 36504423

[B20] Compston J. E. McClung M. R. Leslie W. D. (2019). Osteoporosis. Lancet 393 (10169), 364–376. 10.1016/S0140-6736(18)32112-3 30696576

[B21] Cui Y. Guo Y. Kong L. Shi J. Liu P. Li R. (2021). A bone-targeted engineered exosome platform delivering siRNA to treat osteoporosis. Bioact. Mater. 10, 207–221. 10.1016/j.bioactmat.2021.09.015 34901540 PMC8636739

[B22] Dahl S. L. Rucker R. B. Niklason L. E. (2005). Effects of copper and cross-linking on the extracellular matrix of tissue-engineered arteries. Cell. Transpl. 14 (6), 367–374. 10.3727/000000005783982936 16180655

[B23] Ding H. Gao Y. S. Wang Y. Hu C. Sun Y. Zhang C. (2014). Dimethyloxaloylglycine increases the bone healing capacity of adipose-derived stem cells by promoting osteogenic differentiation and angiogenic potential. Stem Cells Dev. 23 (9), 990–1000. 10.1089/scd.2013.0486 24328551 PMC3996975

[B24] Fan Y. Ni S. Zhang H. (2022). Associations of copper intake with bone mineral density and osteoporosis in adults: data from the national health and nutrition examination survey. Biol. Trace Elem. Res. 200 (5), 2062–2068. 10.1007/s12011-021-02845-5 34283365

[B25] Fan J. Liu Q. Chen T. Chen Y. Wu J. (2024a). Identification of cuproptosis-related genes related to the progression of ankylosing spondylitis by integrated bioinformatics analysis. Med. Baltim. 103 (35), e38313. 10.1097/MD.0000000000038313 PMC11365630 39213249

[B26] Fan X. Sun A. R. Young R. S. E. Afara I. O. Hamilton B. R. Ong L. J. Y. (2024b). Spatial analysis of the osteoarthritis microenvironment: techniques, insights, and applications. Bone Res. 12 (1), 7. 10.1038/s41413-023-00304-6 38311627 PMC10838951

[B27] Feng J. Wang J. Xu Y. Lu F. Zhang J. Han X. (2023). Construction and validation of a novel cuproptosis-mitochondrion prognostic model related with tumor immunity in osteosarcoma. PLoS One 18 (7), e0288180. 10.1371/journal.pone.0288180 37405988 PMC10321638

[B28] Gao Z. Chen S. Ye W. (2025a). Cuproptosis related lncRNA signature as a prognostic and therapeutic biomarker in osteosarcoma immunity. Sci. Rep. 15 (1), 221. 10.1038/s41598-024-84024-9 39747262 PMC11696132

[B29] Gao S. H. Zhu H. R. Chen H. S. Lu H. Wen M. Fan Y. (2025b). Activation of PI3K-AKT pathway prevents steroid-induced osteonecrosis of the femoral head *via* inhibiting cuproptosis. Sci. Rep. 15 (1), 8950–8950. 10.1038/s41598-025-93555-8 40089548 PMC11910512

[B30] GBD 2021 Low Back Pain Collaborators (2023). Global, regional, and national burden of low back pain, 1990-2020, its attributable risk factors, and projections to 2050: a systematic analysis of the global burden of disease study 2021. Lancet Rheumatol. 5 (6), e316–e329. 10.1016/S2665-9913(23)00098-X 37273833 PMC10234592

[B31] Ghanbari M. Salavati-Niasari M. Mohandes F. Dolatyar B. Zeynali B. (2021). *In vitro* study of alginate-gelatin scaffolds incorporated with silica NPs as injectable, biodegradable hydrogels. RSC Adv. 11 (27), 16688–16697. 10.1039/d1ra02744a 35479165 PMC9032273

[B32] Global Burden of Disease 2019 Cancer Collaboration, Kocarnik J. M. Compton K. Dean F. E. Fu W. Gaw B. L. (2022). Cancer incidence, mortality, years of life lost, years lived with disability, and disability-adjusted life years for 29 cancer groups from 2010 to 2019: a systematic analysis for the global burden of disease study 2019. JAMA Oncol. 8 (3), 420–444. 10.1001/jamaoncol.2021.6987 34967848 PMC8719276

[B33] Glyn-Jones S. Palmer A. J. Agricola R. Price A. J. Vincent T. L. Weinans H. (2015). Osteoarthr. Lancet 386 (9991), 376–387. 10.1016/S0140-6736(14)60802-3 25748615

[B34] Gu L. Sun Y. Bai T. Shao S. Tang S. Xue P. (2025). Functional nanozyme system for synergistic tumor immunotherapy *via* cuproptosis and ferroptosis activation. J. Nanobiotechnology 23 (1), 212. 10.1186/s12951-025-03284-3 40089774 PMC11909888

[B35] Guan T. Wu Z. Xu C. Su G. (2023). The association of trace elements with arthritis in US adults: NHANES 2013-2016. J. Trace Elem. Med. Biol. 76, 127122. 10.1016/j.jtemb.2022.127122 36525916

[B36] Gür A. Colpan L. Nas K. Cevik R. Saraç J. Erdoğan F. (2002). The role of trace minerals in the pathogenesis of postmenopausal osteoporosis and a new effect of calcitonin. J. Bone Min. Metab. 20 (1), 39–43. 10.1007/s774-002-8445-y 11810415

[B37] Han Y. L. Luo D. Habaxi K. Tayierjiang J. Zhao W. Wang W. (2022). COL5A2 inhibits the TGF-β and Wnt/β-Catenin signaling pathways to inhibit the invasion and metastasis of osteosarcoma. Front. Oncol. 12, 813809. 10.3389/fonc.2022.813809 35280775 PMC8907856

[B38] Han J. Luo J. Wang C. Kapilevich L. Zhang X. A. (2024). Roles and mechanisms of copper homeostasis and cuproptosis in osteoarticular diseases. Biomed. Pharmacother. 174, 116570. 10.1016/j.biopha.2024.116570 38599063

[B39] Hartvigsen J. Hancock M. J. Kongsted A. Louw Q. Ferreira M. L. Genevay S. (2018). What low back pain is and why we need to pay attention. Lancet 391 (10137), 2356–2367. 10.1016/S0140-6736(18)30480-X 29573870

[B40] Hu H. Dou X. Hu X. Wang L. Ma Y. Liu J. (2023a). Identification of a novel cuproptosis-related gene signature for rheumatoid arthritis-A prospective study. J. Gene Med. 25 (11), e3535. 10.1002/jgm.3535 37338187

[B41] Hu Y. Wang Y. Liu S. Wang H. (2023b). The potential roles of ferroptosis in pathophysiology and treatment of musculoskeletal diseases-opportunities, challenges, and perspectives. J. Clin. Med. 12 (6), 2125. 10.3390/jcm12062125 36983130 PMC10051297

[B42] Hu H. Yin Y. Jiang B. Feng Z. Cai T. Wu S. (2023c). Cuproptosis signature and PLCD3 predicts immune infiltration and drug responses in osteosarcoma. Front. Oncol. 13, 1156455. 10.3389/fonc.2023.1156455 37007130 PMC10060837

[B43] Huang L. Jin W. Bao Y. Zeng X. Zhang Y. Zhou J. (2023). Identification and validation of long noncoding RNA AC083900.1 and RP11-283C24.1 for prediction of progression of osteosarcoma. Mutat. Res. 827, 111828. 10.1016/j.mrfmmm.2023.111828 37437507

[B44] Huang M. Zhang Y. Liu X. (2024). The mechanism of cuproptosis in Parkinson's disease. Ageing Res. Rev. 95, 102214. 10.1016/j.arr.2024.102214 38311254

[B45] Institute of Medicine (US) Panel on Micronutrients (2001). Dietary Reference Intakes for Vitamin A, Vitamin K, Arsenic, Boron, Chromium, Copper, Iodine, Iron, Manganese, Molybdenum, Nickel, Silicon, Vanadium, and Zinc. Washington (DC): National Academies Press, US. 25057538

[B46] Jia C. Liu M. Yao L. Zhao F. Liu S. Li Z. (2024). Multi-omics analysis reveals cuproptosis and mitochondria-based signature for assessing prognosis and immune landscape in osteosarcoma. Front. Immunol. 14, 1280945. 10.3389/fimmu.2023.1280945 38250070 PMC10796547

[B47] Jiang J. Chu D. Lai X. Liu L. Tao J. (2022). The Cuproptosis-Related long noncoding RNA signature predicts prognosis and tumour immune analysis in osteosarcoma. Comput. Math. Methods Med. 2022, 6314182. 10.1155/2022/6314182 36388161 PMC9646308

[B48] Jiang J. Zhan X. Wei J. Fan Q. Li H. Li H. (2023). Artificial intelligence reveals dysregulation of osteosarcoma and cuproptosis-related biomarkers, PDHA1, CDKN2A and neutrophils. Sci. Rep. 13 (1), 4927. 10.1038/s41598-023-32195-2 36967449 PMC10040405

[B49] Kaler S. G. (2011). ATP7A-related copper transport diseases-emerging concepts and future trends. Nat. Rev. Neurol. 7 (1), 15–29. 10.1038/nrneurol.2010.180 21221114 PMC4214867

[B50] Karner C. M. Esen E. Okunade A. L. Patterson B. W. Long F. (2015). Increased glutamine catabolism mediates bone anabolism in response to WNT signaling. J. Clin. Invest. 125 (2), 551–562. 10.1172/JCI78470 25562323 PMC4319407

[B51] Kim B. E. Nevitt T. Thiele D. J. (2008). Mechanisms for copper acquisition, distribution and regulation. Nat. Chem. Biol. 4 (3), 176–185. 10.1038/nchembio.72 18277979

[B52] Kim J. H. Jeon J. Shin M. Won Y. Lee M. Kwak J. S. (2014). Regulation of the catabolic cascade in osteoarthritis by the zinc-ZIP8-MTF1 axis. Cell 156 (4), 730–743. 10.1016/j.cell.2014.01.007 24529376

[B53] León I. E. Porro V. Astrada S. Egusquiza M. G. Cabello C. I. Bollati-Fogolin M. (2014). Polyoxometalates as antitumor agents: bioactivity of a new polyoxometalate with copper on a human osteosarcoma model. Chem. Biol. Interact. 222, 87–96. 10.1016/j.cbi.2014.10.012 25451568

[B54] Li Y. Q. Yin J. Y. Liu Z. Q. Li X. P. (2018). Copper efflux transporters ATP7A and ATP7B: novel biomarkers for platinum drug resistance and targets for therapy. IUBMB Life 70 (3), 183–191. 10.1002/iub.1722 29394468

[B55] Li M. Song Q. Bai Y. Hua F. Wu T. Liu J. (2022). Comprehensive analysis of cuproptosis in immune response and prognosis of osteosarcoma. Front. Pharmacol. 13, 992431. 10.3389/fphar.2022.992431 36263140 PMC9573992

[B56] Li X. Chen X. Gao X. (2023a). Copper and cuproptosis: new therapeutic approaches for Alzheimer's disease. Front. Aging Neurosci. 15, 1300405. 10.3389/fnagi.2023.1300405 38178962 PMC10766373

[B57] Li D. Gao Z. Li Q. Liu X. Liu H. (2023b). Cuproptosis-a potential target for the treatment of osteoporosis. Front. Endocrinol. (Lausanne) 14, 1135181. 10.3389/fendo.2023.1135181 37214253 PMC10196240

[B58] Li Y. Kong C. Wang W. Hu F. Chen X. Xu B. (2023c). Screening of miR-15a-5p as a potential biomarker for intervertebral disc degeneration through RNA-sequencing. Int. Immunopharmacol. 123, 110717. 10.1016/j.intimp.2023.110717 37597405

[B59] Li Q. Fang J. Liu K. Luo P. Wang X. (2024). Multi-omic validation of the cuproptosis-sphingolipid metabolism network: modulating the immune landscape in osteosarcoma. Front. Immunol. 15, 1424806. 10.3389/fimmu.2024.1424806 38983852 PMC11231095

[B60] Li P. Gao Y. Liu W. (2025). Cuproptosis in stroke: molecular mechanisms and therapeutic targeting of copper-mediated cell death. Brain Res. Bull. 232, 111614. 10.1016/j.brainresbull.2025.111614 41183713

[B61] Lin J. Tong X. Shi Z. Zhang D. Zhang L. Wang K. (2020a). A biodegradable Zn-1Cu-0.1Ti alloy with antibacterial properties for orthopedic applications. Acta Biomater. 106, 410–427. 10.1016/j.actbio.2020.02.017 32068137

[B62] Lin W. Xu L. Li G. (2020b). Molecular insights into lysyl oxidases in cartilage regeneration and rejuvenation Front. Bioeng. Biotechnol. 8, 598323. 10.3389/fbioe.2020.598323 32426343 PMC7204390

[B63] Ling W. Li S. Zhu Y. Wang X. Jiang D. Kang B. (2025). Inducers of autophagy and cell death: focus on copper metabolism. Ecotoxicol. Environ. Saf. 290, 117725. 10.1016/j.ecoenv.2025.117725 39823670

[B64] Liu N. Chen M. (2024). Crosstalk between ferroptosis and cuproptosis: from mechanism to potential clinical application. Biomed. Pharmacother. 171, 116115. 10.1016/j.biopha.2023.116115 38181713

[B65] Liu B. Liu Z. Feng C. Li C. Zhang H. Li Z. (2022). Identification of cuproptosis-related lncRNA prognostic signature for osteosarcoma. Front. Endocrinol. (Lausanne) 13, 987942. 10.3389/fendo.2022.987942 36313774 PMC9606239

[B66] Mandell J. B. Lu F. Fisch M. Beumer J. H. Guo J. Watters R. J. (2019). Combination therapy with disulfiram, copper, and doxorubicin for osteosarcoma: *in vitro* support for a novel drug repurposing strategy. Sarcoma 2019, 1320201. 10.1155/2019/1320201 31379466 PMC6657614

[B67] Manolagas S. C. (2010). From estrogen-centric to aging and oxidative stress: a revised perspective of the pathogenesis of osteoporosis. Endocr. Rev. 31 (3), 266–300. 10.1210/er.2009-0024 20051526 PMC3365845

[B68] Matsuoka K. Bakiri L. Wolff L. I. Linder M. Mikels-Vigdal A. Patiño-García A. (2020). Wnt signaling and Loxl2 promote aggressive osteosarcoma. Cell. Res. 30 (10), 885–901. 10.1038/s41422-020-0370-1 32686768 PMC7608146

[B69] Mauro D. Thomas R. Guggino G. Lories R. Brown M. A. Ciccia F. (2021). Ankylosing spondylitis: an autoimmune or autoinflammatory disease? Nat. Rev. Rheumatol. 17 (7), 387–404. 10.1038/s41584-021-00625-y 34113018

[B70] Melnychuk N. Egloff S. Runser A. Reisch A. Klymchenko A. S. (2020). Light-harvesting nanoparticle probes for FRET-based detection of oligonucleotides with single-molecule sensitivity. Angew. Chem. Int. Ed. Engl. 59 (17), 6811–6818. 10.1002/anie.201913804 31943649

[B71] Meyer F. Dittmann A. Kornak U. Herbster M. Pap T. Lohmann C. H. (2021). Chondrocytes from osteoarthritic and chondrocalcinosis cartilage represent different phenotypes. Front. Cell. Dev. Biol. 9, 622287. 10.3389/fcell.2021.622287 33981699 PMC8107373

[B72] Milkovic L. Hoppe A. Detsch R. Boccaccini A. R. Zarkovic N. (2014). Effects of Cu-doped 45S5 bioactive glass on the lipid peroxidation-associated growth of human osteoblast-like cells *in vitro* . J. Biomed. Mater Res. A 102 (10), 3556–3561. 10.1002/jbm.a.35032 24243858

[B73] Ni S. Hong J. Li W. Ye M. Li J. (2023). Construction of a cuproptosis-related lncRNA signature for predicting prognosis and immune landscape in osteosarcoma patients. Cancer Med. 12 (4), 5009–5024. 10.1002/cam4.5214 36129020 PMC9972154

[B74] Nong J. Lu G. Huang Y. Liu J. Chen L. Pan H. (2023). Identification of cuproptosis-related subtypes, characterization of immune microenvironment infiltration, and development of a prognosis model for osteoarthritis. Front. Immunol. 14, 1178794. 10.3389/fimmu.2023.1178794 37809099 PMC10551149

[B75] Nunes E. A. Stokes T. McKendry J. Currier B. S. Phillips S. M. (2022). Disuse-induced skeletal muscle atrophy in disease and nondisease states in humans: mechanisms, prevention, and recovery strategies. Am. J. Physiology-Cell Physiology, 322 (6), C1068–C1084. 10.1152/ajpcell.00425.2021 35476500

[B76] Okyay E. Ertugrul C. Acar B. Sisman A. R. Onvural B. Ozaksoy D. (2013). Comparative evaluation of serum levels of main minerals and postmenopausal osteoporosis. Maturitas 76 (4), 320–325. 10.1016/j.maturitas.2013.07.015 24011991

[B77] Olivares M. Uauy R. (1996). Limits of metabolic tolerance to copper and biological basis for present recommendations and regulations. Am. J. Clin. Nutr. 63 (5), 846S–852S. 10.1093/ajcn/63.5.846 8615373

[B78] Omoto A. Kawahito Y. Prudovsky I. Tubouchi Y. Kimura M. Ishino H. (2005). Copper chelation with tetrathiomolybdate suppresses adjuvant-induced arthritis and inflammation-associated cachexia in rats. Arthritis Res. Ther. 7 (6), R1174–R1182. 10.1186/ar1801 16277669 PMC1297562

[B79] Oryan A. Sahvieh S. (2021). Effects of bisphosphonates on osteoporosis: focus on zoledronate. Life Sci. 264, 118681. 10.1016/j.lfs.2020.118681 33129881

[B80] Pan Y. Ai C. X. Zeng L. Liu C. Li W. C. (2020). Modulation of copper-induced antioxidant defense, Cu transport, and mitophagy by hypoxia in the large yellow croaker (Larimichthys crocea). Fish. Physiol. Biochem. 46 (3), 997–1010. 10.1007/s10695-020-00765-0 31925663

[B81] Pasqualicchio M. Gasperini R. Velo G. P. Davies M. E. (1996). Effects of copper and zinc on proteoglycan metabolism in articular cartilage. Mediat. Inflamm. 5 (2), 95–99. 10.1155/S0962935196000154 18475704 PMC2365780

[B82] Pepa G. D. Brandi M. L. (2016). Microelements for bone boost: the last but not the least. Clin. Cases Min. Bone Metab. 13 (3), 181–185. 10.11138/ccmbm/2016.13.3.181 28228778 PMC5318168

[B83] Petek D. Hannouche D. Suvà D. (2019). Osteonecrosis of the femoral head: pathophysiology and current concepts of treatment. EFORT Open Rev. 4 (3), 85–97. 10.1302/2058-5241.4.180036 30993010 PMC6440301

[B84] Qu X. He Z. Qiao H. Zhai Z. Mao Z. Yu Z. (2018). Serum copper levels are associated with bone mineral density and total fracture. J. Orthop. Transl. 14, 34–44. 10.1016/j.jot.2018.05.001 30035031 PMC6034109

[B85] Rodriguez-Pascual F. Rosell-Garcia T. (2018). Lysyl oxidases: functions and disorders. J. Glaucoma 27 (Suppl. 1), S15–S19. 10.1097/IJG.0000000000000910 29419646

[B86] Rondanelli M. Faliva M. A. Infantino V. Gasparri C. Iannello G. Perna S. (2021). Copper as dietary supplement for bone metabolism: a review. Nutrients 13 (7), 2246. 10.3390/nu13072246 34210051 PMC8308383

[B87] Rucker R. B. Kosonen T. Clegg M. S. Mitchell A. E. Rucker B. R. Uriu-Hare J. Y. (1998). Copper, lysyl oxidase, and extracellular matrix protein cross-linking. Am. J. Clin. Nutr. 67 (5l), 996S–1002S. 10.1093/ajcn/67.5.996S 9587142

[B88] Scudder P. R. McMurray W. White A. G. Dormandy T. L. (1978). Synovial fluid copper and related variables in rheumatoid and degenerative arthritis. Ann. Rheum. Dis. 37 (1), 71–72. 10.1136/ard.37.1.71 629608 PMC1000193

[B89] Shi P. Cheng Z. Zhao K. Chen Y. Zhang A. Gan W. (2023). Active targeting schemes for nano-drug delivery systems in osteosarcoma therapeutics. J. Nanobiotechnology 21 (1), 103. 10.1186/s12951-023-01826-1 36944946 PMC10031984

[B90] Smolen J. S. Aletaha D. McInnes I. B. (2016). Rheumatoid arthritis Lancet 388 (10055), 2023–2038. 10.1016/S0140-6736(16)30173-8 27156434

[B91] Sokolov A. V. Acquasaliente L. Kostevich V. A. Frasson R. Zakharova E. T. Pontarollo G. (2015). Thrombin inhibits the anti-myeloperoxidase and ferroxidase functions of ceruloplasmin: relevance in rheumatoid arthritis. Free Radic. Biol. Med. 86, 279–294. 10.1016/j.freeradbiomed.2015.05.016 26001728

[B92] Staszkiewicz R. Sobański D. Ulasavets U. Wieczorek J. Golec E. Marcol W. (2023). Evaluation of the concentration of selected elements in serum patients with intervertebral disc degeneration. J. Trace Elem. Med. Biol. 77, 127145. 10.1016/j.jtemb.2023.127145 36921371

[B93] Stegen S. Stockmans I. Moermans K. Thienpont B. Maxwell P. H. Carmeliet P. (2018). Osteocytic oxygen sensing controls bone mass through epigenetic regulation of sclerostin. Nat. Commun. 9 (1), 2557. 10.1038/s41467-018-04679-7 29967369 PMC6028485

[B94] Sun Y. Chen P. Zhao B. (2024). Role of extracellular vesicles associated with microRNAs and their interplay with cuproptosis in osteoporosis. Noncoding RNA Res. 9 (3), 715–719. 10.1016/j.ncrna.2024.03.002 38577024 PMC10990744

[B95] Sun H. Zou Y. Chen Z. He Y. Ye K. Liu H. (2025). Nanodrug-engineered exosomes achieve a jointly dual-pathway inhibition on cuproptosis. Adv. Sci. (Weinh) 12 (4), e2413408. 10.1002/advs.202413408 39639737 PMC11775538

[B96] Taipaleenmäki H. Saito H. Schröder S. Maeda M. Mettler R. Ring M. (2022). Antagonizing microRNA-19a/b augments PTH anabolic action and restores bone mass in osteoporosis in mice. EMBO Mol. Med. 14 (11), e13617. 10.15252/emmm.202013617 36193848 PMC9641424

[B97] Tang D. Chen X. Kroemer G. (2022). Cuproptosis: a copper-triggered modality of mitochondrial cell death. Cell. Res. 32 (5), 417–418. 10.1038/s41422-022-00653-7 35354936 PMC9061796

[B98] Tong X. Tang R. Xiao M. Xu J. Wang W. Zhang B. (2022). Targeting cell death pathways for cancer therapy: recent developments in necroptosis, pyroptosis, ferroptosis, and cuproptosis research. J. Hematol. Oncol. 15 (1), 174. 10.1186/s13045-022-01392-3 36482419 PMC9733270

[B99] Tsitkanou S. Della Gatta P. A. Russell A. P. (2016). Skeletal muscle satellite cells, mitochondria, and MicroRNAs: their involvement in the pathogenesis of ALS. Front. Physiol. 7, 403. 10.3389/fphys.2016.00403 27679581 PMC5020084

[B100] Tsvetkov P. Coy S. Petrova B. Dreishpoon M. Verma A. Abdusamad M. (2022). Copper induces cell death by targeting lipoylated TCA cycle proteins. Science 375 (6586), 1254–1261. 10.1126/science.abf0529 35298263 PMC9273333

[B101] Turnlund J. R. Keyes W. R. Peiffer G. L. Scott K. C. (1998). Copper absorption, excretion, and retention by young men consuming low dietary copper determined by using the stable isotope 65Cu. Am. J. Clin. Nutr. 67 (6), 1219–1225. 10.1093/ajcn/67.6.1219 9625096

[B102] Voshtani R. Hou P. Liu Z. Cao L. Feng C. Shao C. (2025). IGF2 regulates proliferation, differentiation, and mitochondrial bioenergetics in human satellite cells. Biol. Direct, 20 (1), 115–115. 10.1186/s13062-025-00716-w 41430340 PMC12750578

[B103] Walsh J. A. Magrey M. (2021). Clinical manifestations and diagnosis of axial spondyloarthritis. J. Clin. Rheumatol. 27 (8), e547–e560. 10.1097/RHU.0000000000001575 33105312 PMC8612900

[B104] Wang Y. Yao Y. Thirumurugan M. Prabakaran S. Rajan M. Wang K. (2021). Natural drug-loaded bimetal-substituted hydroxyapatite-polymeric composite for osteosarcoma-affected bone repair. Front. Cell. Dev. Biol. 9, 731887. 10.3389/fcell.2021.731887 34616738 PMC8488211

[B105] Wang C. Guo S. Gu Q. Wang X. Long L. Xiao C. (2022a). Exosomes: a promising therapeutic strategy for intervertebral disc degeneration. Exp. Gerontol. 163, 111806. 10.1016/j.exger.2022.111806 35417774

[B106] Wang Y. Zhang L. Zhou F. (2022b). Cuproptosis: a new form of programmed cell death. Cell. Mol. Immunol. 19 (8), 867–868. 10.1038/s41423-022-00866-1 35459854 PMC9338229

[B107] Wang X. Xie C. Lin L. (2022c). Development and validation of a cuproptosis-related lncRNA model correlated to the cancer-associated fibroblasts enable the prediction prognosis of patients with osteosarcoma. J. Bone Oncol. 38, 100463. 10.1016/j.jbo.2022.100463 36569351 PMC9772846

[B108] Wang M. Zheng L. Ma S. Lin R. Li J. Yang S. (2023a). Cuproptosis: emerging biomarkers and potential therapeutics in cancers. Front. Oncol. 13, 1288504. 10.3389/fonc.2023.1288504 38023234 PMC10662309

[B109] Wang D. Tian Z. Zhang P. Zhen L. Meng Q. Sun B. (2023b). The molecular mechanisms of cuproptosis and its relevance to cardiovascular disease. Biomed. Pharmacother. 163, 114830. 10.1016/j.biopha.2023.114830 37150036

[B110] Wang W. Chen Z. Hua Y. (2023c). Bioinformatics prediction and experimental validation identify a novel Cuproptosis-Related gene signature in human synovial inflammation during osteoarthritis progression. Biomolecules 13 (1), 127. 10.3390/biom13010127 36671512 PMC9855951

[B111] Wang A. Liu W. Jin Y. Wei B. Fan Y. Guo X. (2023d). Identification of immunological characteristics and cuproptosis-related molecular clusters in rheumatoid arthritis. Int. Immunopharmacol. 123, 110804. 10.1016/j.intimp.2023.110804 37595490

[B112] Wang W. Lu K. Jiang X. Wei Q. Zhu L. Wang X. (2023e). Ferroptosis inducers enhanced cuproptosis induced by copper ionophores in primary liver cancer. J. Exp. Clin. Cancer Res. 42 (1), 142. 10.1186/s13046-023-02720-2 37277863 PMC10242978

[B113] Wang C. Gong S. Liu H. Cui L. Ye Y. Liu D. (2024). Angiogenesis unveiled: insights into its role and mechanisms in cartilage injury. Exp. Gerontol. 195, 112537. 10.1016/j.exger.2024.112537 39111547

[B114] Wei B. Wang S. Li S. Gu Q. Yue Q. Tang Z. (2025). Unveiling Cuproptosis-Driven molecular clusters and immune dysregulation in ankylosing spondylitis. J. Inflamm. Res. 18, 863–882. 10.2147/JIR.S502520 39867949 PMC11760765

[B115] Xia J. Hu C. Ji Y. Wang M. Jin Y. Ye L. (2023). Copper-loaded nanoheterojunction enables superb orthotopic osteosarcoma therapy *via* oxidative stress and cell cuproptosis. ACS Nano 17 (21), 21134–21152. 10.1021/acsnano.3c04903 37902237

[B116] Xie J. Yang Y. Gao Y. He J. (2023). Cuproptosis: mechanisms and links with cancers. Mol. Cancer 22 (1), 46. 10.1186/s12943-023-01732-y 36882769 PMC9990368

[B117] Xie C. Sun Q. Chen J. Yang B. Lu H. Liu Z. (2024). Cu-Tremella fuciformis polysaccharide-based tumor microenvironment-responsive injectable gels for cuproptosis-based synergistic osteosarcoma therapy. Int. J. Biol. Macromol. 270 (Pt 2), 132029. 10.1016/j.ijbiomac.2024.132029 38704064

[B118] Yan J. Xia D. Zhou W. Li Y. Xiong P. Li Q. (2020). pH-responsive silk fibroin-based CuO/Ag micro/nano coating endows polyetheretherketone with synergistic antibacterial ability, osteogenesis, and angiogenesis. Acta Biomater. 115, 220–234. 10.1016/j.actbio.2020.07.062 32777292

[B119] Yan H. Shi L. Li Y. Zhang Z. (2025). Decoding potential Cuproptosis-Related genes in sarcopenia: a multi-omics network analysis. Biology, 14 (12), 1642. 10.3390/biology14121642 41463417 PMC12729620

[B120] Yang M. Zheng H. Xu K. Yuan Q. Aihaiti Y. Cai Y. (2022a). A novel signature to guide osteosarcoma prognosis and immune microenvironment: cuproptosis-related lncRNA. Front. Immunol. 13, 919231. 10.3389/fimmu.2022.919231 35967366 PMC9373797

[B121] Yang W. Wu H. Tong L. Wang Y. Guo Q. Xu L. (2022b). A cuproptosis-related genes signature associated with prognosis and immune cell infiltration in osteosarcoma. Front. Oncol. 12, 1015094. 10.3389/fonc.2022.1015094 36276092 PMC9582135

[B122] Yang W. M. Lv J. F. Wang Y. Y. Xu Y. M. Lin J. Liu J. (2023). The daily intake levels of copper, selenium, and zinc are associated with osteoarthritis but not with rheumatoid arthritis in a cross-sectional study. Biol. Trace Elem. Res. 201 (12), 5662–5670. 10.1007/s12011-023-03636-w 36943549

[B123] Yazar M. Sarban S. Kocyigit A. Isikan U. E. (2005). Synovial fluid and plasma selenium, copper, zinc, and iron concentrations in patients with rheumatoid arthritis and osteoarthritis. Biol. Trace Elem. Res. 106 (2), 123–132. 10.1385/BTER:106:2:123 16116244

[B124] Yellowley C. E. Genetos D. C. (2019). Hypoxia signaling in the skeleton: implications for bone health. Curr. Osteoporos. Rep. 17 (1), 26–35. 10.1007/s11914-019-00500-6 30725321 PMC6653634

[B125] Yoshioka J. Lee R. (2013). Thioredoxin-interacting protein and myocardial mitochondrial function in ischemia–reperfusion injury. Trends Cardiovasc. Med., 24 (2), 75–80. 10.1016/j.tcm.2013.06.007 23891554 PMC3870036

[B126] Yu S. Yao X. (2024). Advances on immunotherapy for osteosarcoma. Mol. Cancer 23 (1), 192. 10.1186/s12943-024-02105-9 39245737 PMC11382402

[B127] Yu Q. Xiao Y. Guan M. Zhang X. Yu J. Han M. (2024). Copper metabolism in osteoarthritis and its relation to oxidative stress and ferroptosis in chondrocytes. Front. Mol. Biosci. 11, 1472492. 10.3389/fmolb.2024.1472492 39329090 PMC11425083

[B128] Zhang P. He J. Gan Y. Shang Q. Chen H. Zhao W. (2023). Unravelling diagnostic clusters and immune landscapes of cuproptosis patterns in intervertebral disc degeneration through dry and wet experiments. Aging (Albany NY). 15 (24), 15599–15623. 10.18632/aging.205449 38159257 PMC10781477

[B129] Zhang P. Chen H. Zhang Y. Liu Y. Zhu G. Zhao W. (2024a). Dry and wet experiments reveal diagnostic clustering and immune landscapes of cuproptosis patterns in patients with ankylosing spondylitis. Int. Immunopharmacol. 127, 111326. 10.1016/j.intimp.2023.111326 38091828

[B130] Zhang Y. Zhang N. Xing J. Sun Y. Jin X. Shen C. (2024b). *In situ* hydrogel based on Cu-Fe3O4 nanoclusters exploits oxidative stress and the ferroptosis/cuproptosis pathway for chemodynamic therapy. Biomaterials 311, 122675. 10.1016/j.biomaterials.2024.122675 38943822

[B131] Zhao J. Guo S. Schrodi S. J. He D. (2022). Cuproptosis and cuproptosis-related genes in rheumatoid arthritis: implication, prospects, and perspectives. Front. Immunol. 13, 930278. 10.3389/fimmu.2022.930278 35990673 PMC9386151

[B132] Zhou H. W. Lou S. Q. Zhang K. (2004). Recovery of function in osteoarthritic chondrocytes induced by p16INK4a-specific siRNA *in vitro* . Rheumatol. Oxf. 43 (5), 555–568. 10.1093/rheumatology/keh127 15026580

[B133] Zhou J. Liu C. Sun Y. Francis M. Ryu M. S. Grider A. (2021). Genetically predicted circulating levels of copper and zinc are associated with osteoarthritis but not with rheumatoid arthritis. Osteoarthr. Cartil. 29 (7), 1029–1035. 10.1016/j.joca.2021.02.564 33640581

[B134] Zhou H. Qian Q. Chen Q. Chen T. Wu C. Chen L. (2024). Enhanced mitochondrial targeting and inhibition of pyroptosis with multifunctional metallopolyphenol nanoparticles in intervertebral disc degeneration. Small 20 (13), e2308167. 10.1002/smll.202308167 37953455

[B135] Zhu J. Tang Y. Wu Q. Ji Y. C. Kang F. W. (2019). Mechanism of participation of osteocytes in the formation of osteoclasts under hypoxia. Hua Xi Kou Qiang Yi Xue Za Zhi 37 (5), 463–468. 10.7518/hxkq.2019.05.002 31721490 PMC7030417

[B136] Zhu C. Han S. Zeng X. Zhu C. Pu Y. Sun Y. (2022). Multifunctional thermo-sensitive hydrogel for modulating the microenvironment in osteoarthritis by polarizing macrophages and scavenging RONS. J. Nanobiotechnology 20 (1), 221. 10.1186/s12951-022-01422-9 35526013 PMC9077879

[B137] Zofková I. Nemcikova P. Matucha P. (2013). Trace elements and bone health. Clin. Chem. Lab. Med. 51 (8), 1555–1561. 10.1515/cclm-2012-0868 23509220

